# Predicting Protein Function with Hierarchical Phylogenetic Profiles: The Gene3D Phylo-Tuner Method Applied to Eukaryotic Genomes

**DOI:** 10.1371/journal.pcbi.0030237

**Published:** 2007-11-30

**Authors:** Juan A. G Ranea, Corin Yeats, Alastair Grant, Christine A Orengo

**Affiliations:** Department of Biochemistry and Molecular Biology, University College London, London, United Kingdom; Columbia University, United States of America

## Abstract

“Phylogenetic profiling” is based on the hypothesis that during evolution functionally or physically interacting genes are likely to be inherited or eliminated in a codependent manner. Creating presence–absence profiles of orthologous genes is now a common and powerful way of identifying functionally associated genes. In this approach, correctly determining orthology, as a means of identifying functional equivalence between two genes, is a critical and nontrivial step and largely explains why previous work in this area has mainly focused on using presence–absence profiles in prokaryotic species. Here, we demonstrate that eukaryotic genomes have a high proportion of multigene families whose phylogenetic profile distributions are poor in presence–absence information content. This feature makes them prone to orthology mis-assignment and unsuited to standard profile-based prediction methods. Using CATH structural domain assignments from the Gene3D database for 13 complete eukaryotic genomes, we have developed a novel modification of the phylogenetic profiling method that uses genome copy number of each domain superfamily to predict functional relationships. In our approach, superfamilies are subclustered at ten levels of sequence identity—from 30% to 100%—and phylogenetic profiles built at each level. All the profiles are compared using normalised Euclidean distances to identify those with correlated changes in their domain copy number. We demonstrate that two protein families will “auto-tune” with strong co-evolutionary signals when their profiles are compared at the similarity levels that capture their functional relationship. Our method finds functional relationships that are not detectable by the conventional presence–absence profile comparisons, and it does not require a priori any fixed criteria to define orthologous genes.

## Author Summary

The vast number of protein sequences being determined by the international genomics projects means that it is not possible to functionally characterise all the proteins through direct experimentation. One of the more successful electronic methods for detecting functionally associated genes has been through the comparison of genes' phylogenetic profiles. This method is based on the hypothesis that two functionally related genes will show very similar presence–absence profile patterns throughout different organisms. Whilst these methods have grown increasingly sophisticated, they have largely been based on detecting functionally homologous genes in different species (technically known as orthologous genes) and thus better suited to prokaryotic genomes, where this can be done more easily. We have developed a new type of hierarchical phylogenetic profile by subdividing protein families into subclusters in different sequence identity levels. This new approach encapsulates a more realistic model of the functional variation that uneven natural selection pressure produces on different protein families and organisms, and it can detect functional relationships between protein families without the initial application of rigid sequence similarity thresholds or complex protocols for orthology assignment. These advantages are especially useful in eukaryotes since the larger average size of eukaryotic multigene families makes them more prone to orthology mis-assignment than in prokaryotes.

## Introduction

Comparison of the phylogenetic profiles of orthologous proteins in different species is a well-known and powerful method for detecting functionally related proteins. The approach assumes that two functionally related proteins will have been inherited or eliminated in a codependent fashion through speciation. Therefore, by examining correlated presence–absence patterns in different genomes, it is possible to infer protein co-evolution and a functional relationship.

After the original idea was published [1], the phylogenetic profile method was improved or reinterpreted in many different ways. For example: through the application of more complex logical rules to associate and compare protein profiles [2]; the use of domain profiles instead of whole proteins [3]; refining the algorithm [4]; or integration of species phylogenetic information [5,6].

Although the phylogenetic profile method can be improved by integrating new sources of information, in all cases the prediction quality of this method depends on two critical steps: the selection of the reference species sample and the determination of which proteins are orthologues. Typically the latter is done using a “Reciprocal Best Hits” (RBH) approach with similarity determined by the BLAST algorithm [4,7–8] and an E-value cutoff for potential orthologues. In fact, these two steps have different impacts on the prediction quality. The reference species problem can be avoided by simply increasing the sample size with new genomes until a certain number has been reached. However, there are many problems, e.g., [8–11], in determining orthology (two genes from two different species that derive from a single gene in the last common ancestor), especially the separation of orthologues from paralogues (genes that derive from a single gene that was duplicated within a genome). Multigene families that exist within one genome can also exhibit functional overlap and substitutability between the members.

The fact that genes evolve at different rates, due to both uneven natural selection pressure on their functions and different species having different mutation rates—e.g., rodents accumulate point mutations more rapidly than apes [12] —implies that the evolutionary rates of proteins may vary over several orders of magnitude in the different gene families [13]. This rate variation makes it difficult to choose a single similarity E-value cutoff that can be broadly applied to identify those orthologues most likely to have retained similar functionality.

The multigene family problem is particularly challenging in eukaryotic genomes wherein the percentage of genes present in multiple homologous copies is much higher than in prokaryotic genomes. However, the higher percentage of multigene families is not the only problem that makes it more difficult to correctly assign orthologous relationships in eukaryotic species. In contrast to prokaryotes, accurate identification of ORFs (open reading frames) is complicated in eukaryotes by noise from domain rearrangements, more complex gene architectures, and a higher presence of noncoding regions. Furthermore, in eukaryotes there is a weaker correlation between the number of ORFs and the phenotypic complexity of an organism. This is probably due to a number of reasons, perhaps most significantly the greater use of RNA-based regulatory mechanisms [14].

We have developed a novel modification of the phylogenetic profile method that bypasses several of these problems, especially the orthology—or functional equivalence as it can also be perceived—detection problem, and can detect interacting multigene families. This method is particularly applicable to identifying functional networks in eukaryotes, which have so far proven intractable.

Our approach is based around protein domains, since these are the most elemental units of protein function. Furthermore, this allows us to bypass confusion caused by domain rearrangements. For this study we have used the domain annotation from the Gene3D database, which stores CATH assignments for complete genomes. The first key modification is that we do not consider the presence–absence of domains but the number of copies of the domain. The second key modification is that we subcluster all the domains at ten levels of sequence identity from 30% to 100%. We then create profiles for every domain family and the subclusters within it, which enables the identification of distinct functional subgroups within domain families.

Although it is clear that there are always exceptions to any evolutionary model that can be proposed, the co-evolutionary hypothesis implicit in our model supposes that gene copy number in two functionally related protein clusters (superfamilies or subclusters) will vary in a related fashion. In our approach, domain occurrence profiles are built at many identity levels, and therefore it is expected that two protein clusters will “auto-tune” with a significant correlation signal when their profiles are compared at the similarity levels that retain their functional relationship. Therefore, domain occurrence profiles were compared all against all (superfamilies and subclusters) to identify correlations in domain copy number variation in all the different identity levels. Our method found strong co-evolutionary signals amongst functionally related multigene domain families that could not have been predicted by the conventional presence–absence comparison of profiles proposed by Pellegrini et al. [1].

This new approach has a number of features that make it especially useful for eukaryotic genome analysis. Firstly, phylogenetic profiles based on protein domains can detect functional relationships that are not detectable using phylogenetic profiles of whole proteins, reducing the noise that protein domain rearrangements produce, particularly in eukaryotes [3]. Secondly, it uses domain occurrence profiles instead of presence–absence profiles. The latter are less effective in eukaryotic genomes as they do not account for the wide variation in gene copy number observed in eukaryotes. And thirdly, the method applied does not require a priori any fixed E-value cutoff to define orthologous groups. Because domain clusters are built at several discrete identity levels, the method takes into account much of the variation that uneven selection pressure produces on sequence and functional conservation.

## Results/Discussion

### Calculating the Information Content of Eukaryotic and Prokaryotic Profiles

**Figure 1:**
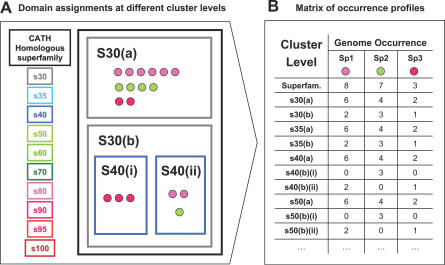
Building Gene3D Phylogenetic Occurrence Profiles (A) Domain family assignments at different cluster levels: the different sequence identity levels are indicated (s30, 30% sequence identity; s35, s40, etc.); circles represent sequences from different species (colours), grouped by sequence identity. (B) Matrix of occurrence profiles for different clusters (superfamilies and their derived sequence identity subclusters). Occurrence profiles are derived from the number of relatives identified in each genome for each sequence identity cluster. Thus phylogenetic profiles are generated for all the clusters (superfamilies and subclusters) across complete genomes in Gene3D.

Using CATH structural domain assignments from the Gene3D database for 13 complete eukaryotic genomes and 106 complete prokaryotic genomes, all superfamily domains found in eukaryotes and prokaryotes were clustered at ten sequence identity levels (from 30% to 100%; see Figure 1). Each domain was hence assigned a unique identifier composed of the four-part CATH code and a ten-part hierarchical cluster code allowing the simple creation of profiles. Occurrence profiles across species were calculated for all the identity and superfamily levels in these two phyla. Subsequently 10,005 eukaryotic and 28,080 prokaryotic profiles with sufficient taxonomic representation (present in six or more species) were selected for further analysis (see Materials and Methods).

To compare the information content associated with eukaryotic and prokaryotic profiles, we performed two kinds of calculations based on different features of the gene distribution in the profiles. One of these calculations is related to the presence–absence pattern of the domain clusters throughout the different species, and it is similar to that used by other groups (e.g., [3]). This will be referred to as R+/− information content. The other measure is related to the variation in gene copy number throughout the species in the profiles, and this will be referred to as Ro or occurrence information (see Materials and Methods).

**Figure 2:**
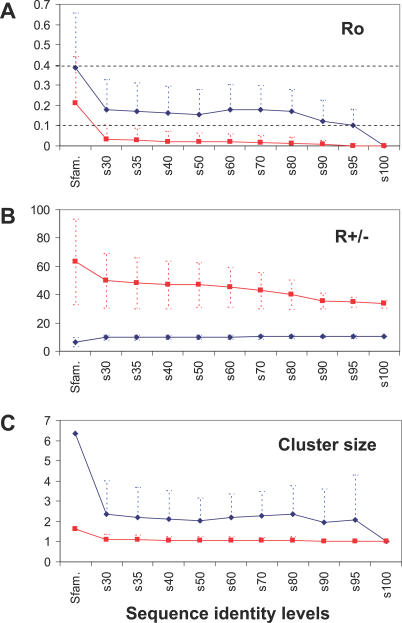
Comparison between Eukaryotic (Blue) and Prokaryotic (Red) (A) Ro and (B) R +/− Information Content Averages at Different Sequence Identity Levels (*x*-Axis); and (C) Average Cluster Size (Average Number of Gene Copies per Species along the *x*-Axis) Standard deviations are also shown (blue and red dashed lines). The large average sizes and standard deviations found at the superfamily level in the cluster sizes plot distorts the graph and therefore the medians of the distributions are shown instead. Ro information value boundaries for eukaryotic profile selection are also indicated (black horizontal dashed lines in (A).

The profiles of the eukaryotic clusters show significantly higher Ro information content values than the prokaryotic profiles for all sequence identity levels except the s100 level (see Figure 2A). In contrast, the R+/− information content in eukaryotic profiles is constantly and significantly less than in prokaryotes (see Figure 2B). These results seem to be explained by the differences in the gene copy distributions and average cluster sizes between the two phyla. Eukaryotic genomes show average cluster sizes of around two gene copies per species, and therefore a higher proportion of multigene clusters than in prokaryotes, whose protein clusters are about one copy per species at all identity levels (see Figure 2C).

Larger multigene clusters in eukaryotes are also related to a larger cluster size variation and Ro information variation in this phylum than in prokaryotes (compare the standard deviation values of Ro and cluster sizes between the two phyla at different sequence identity levels in Figure 2A and 2C). In contrast, lower average cluster sizes of one gene copy per species, distributed throughout a higher number of species, give rise to profiles with lower Ro and higher R+/− information content in the prokaryotic profiles (see Figure 2A and 2B).

These results indicate that except for the s100 level, eukaryotic genomes have a high proportion of multigene families whose phylogenetic profile distributions are poor in R+/− information content. This feature of eukaryotic profiles makes them prone to orthology mis-assignment and bad models for the standard phylogenetic prediction methods of the “1/0 gene presence–absence” type. In contrast, a majority of the eukaryotic profiles show high Ro information content not previously exploited in conventional correlated profiles analyses. There are no prokaryotic profiles with Ro information content above 0.1, except for at the superfamily level (see Figure 2A). Therefore, because we wanted to focus on the analysis of the previously neglected multigene families, without applying conventional 1/0 phylogenetic profile comparisons, only the cluster profiles with Ro ≥ 0.1 were selected for further analysis.

### Removing the Effect of Genome Size Correlation on Profile Comparison in Eukaryotes

In many cases a high correlation amongst multigene families and genome size variation has been observed [15]. This is likely to lead to spurious correlations between occurrence profiles due to their correlation with genome size. To analyse the effect on occurrence profile comparison, correlations with genome size were calculated for all the profiles in the eukaryotic matrix (see Materials and Methods).

**Figure 3:**
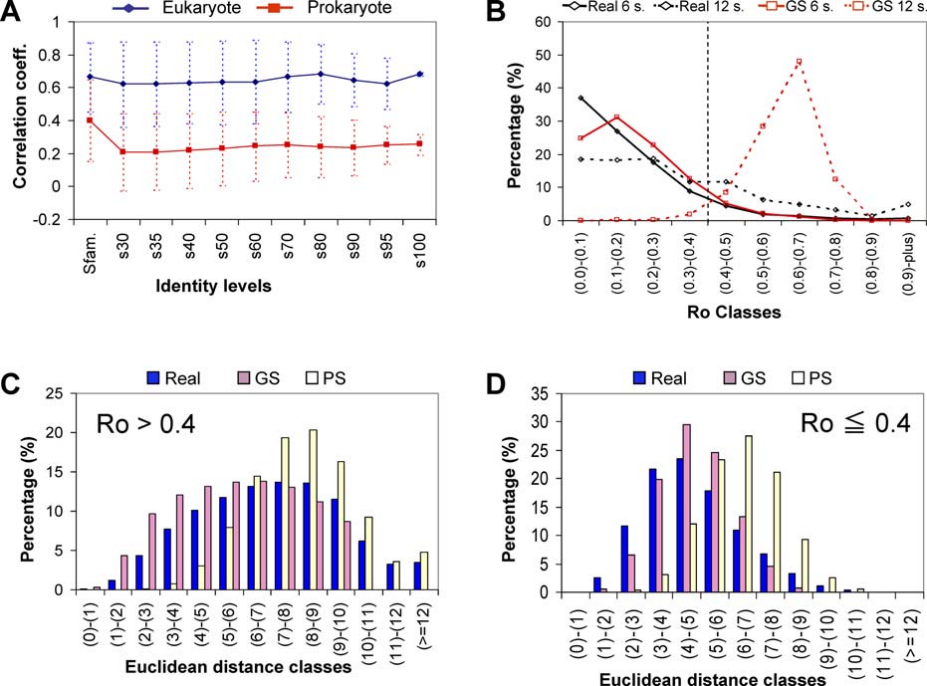
Analysis of the Genome Size Correlation Effect on the Eukaryotic Profile Similarity Calculation (A) Average genome size correlations (*y*-axis) of eukaryotic (blue) and prokaryotic (red) profiles at different sequence identity levels (*x*-axis) and their corresponding standard deviation (vertical dashed lines). (B) Percentage distribution of profiles (*y*-axis) by their Ro information values (*x*-axis), for profiles from the real model (black) and from the GS random model (red), having gene representations in six species (continuous line) and in 12 species (dashed lines); the Ro = 0.4 boundary is also shown (vertical black dashed line). (C,D) Percentage distribution of profile pairs (*y*-axis) with (C) Ro > 0.4 and (D) Ro ≤ 0.4 for different Ed bins (*x*-axis) for the real matrix (blue), and for the GS (pink) and the PS (yellow) models.

At all sequence identity levels, eukaryotic profiles show a bias toward higher genome size correlation values compared to the prokaryotic sample (see Figure 3A). The heterogeneous distribution of genome sizes in different phylogenetic groups for the eukaryotic sample (see Figures S1A and S1B), the larger average genome sizes and the higher proportion of multigene families in this phylogenetic group are all likely to be contributing to the trend for these profiles to correlate with genome size. Whatever the causes, this tendency of eukaryotic profiles to correlate with genome size increases the probability of profile pairs having high similarity scores due to this spurious shared tendency.

To estimate the effect that genome size correlation has on the Euclidean distance (Ed) score used to measure profile similarity (see Material and Methods), the Ro information content and the profiles' Eds were calculated and compared for the random and the real matrix models (see Figure 3B–3D; see Materials and Methods for description of the random and real matrices). Ro information values of the GS (random model—Genome Shuffling) profiles' matrix distribute similarly to those of the real profiles' matrix except for universally distributed clusters (e.g., clusters present in 12 eukaryotic species, see Figure 3B). GS profiles of these universally distributed clusters show a clearly differentiated distribution with significant bias toward higher Ro values than the real cluster profiles (see Figure 3B).

Ed comparison of the profile pairs shows that GS profiles with Ro values above 0.4 contribute most to the error in the similarity calculations associated with genome size correlation (see Figure 3C). This observation is reversed and the GS distribution is shifted to higher distance classes when similarities are calculated for profiles with Ro below 0.4 (see Figure 3D and compare with Figure 3C). Therefore Ro = 0.4 appears to be a good selection boundary to reduce the genome size correlation error on profile similarity calculations. As observed in Figure 2A, this Ro = 0.4 boundary only affects eukaryotic profiles at the lowest sequence identity level and also shows that universal and highly populated superfamilies are more likely to generate spurious similarity scores due to their probable correlation with genome size (as also observed in [15]). Therefore, to reduce the error arising from genome size correlation in profile similarity calculations, profiles with Ro > 0.4 were removed before performing further analyses on the matrix.

### Assessment of Profile Similarity and Predicting Functionally Related Clusters

**Figure 4:**
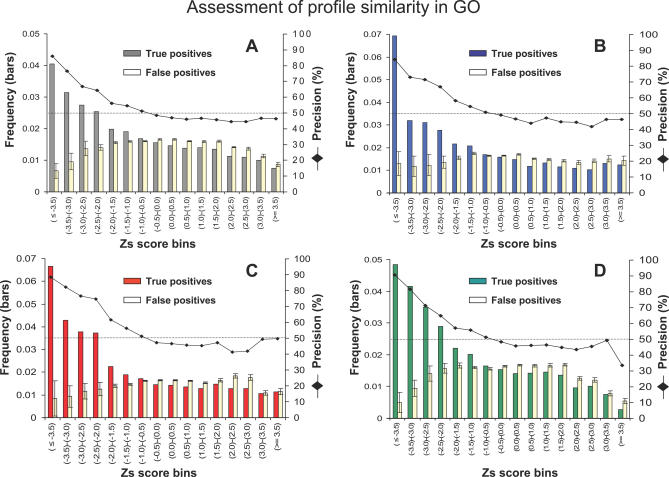
Association of the Zs Similarity Values, for Protein Cluster Comparisons, with Functional Relationships Frequency (left-hand *y*-axis) of TPs and FPs (bars) in different profile similarity Zs bins (*x*-axis) for (A), all functional groups dataset (grey bars); (B), cellular component (blue); (C), molecular function (red) and biological processes (green). FP frequencies (yellow bars) are the average values and standard deviations (vertical lines) calculated from ten different randomised versions of each of the four functionally related clusters' datasets. The method's precision percentage (right-hand *y*-axis) for different Zs bins (*x*-axis) and the random precision 50% value (dashed line) are also shown.

Finally, a matrix of 3,721 different eukaryotic profiles with protein clusters present in at least six out of 13 species and with 0.1 ≤ Ro ≤ 0.4 were selected for profile pair similarity comparison and functional prediction analysis. Z scores (Zs) for the similarity comparisons were calculated from an all-against-all comparison of profile pairs (see Materials and Methods). Functionally related pairs of protein clusters from the four datasets (“cellular components”, “biological processes”, “biological function”, and the “all functional groups” dataset; see Materials and Methods) were identified within the whole dataset of profile pairs, and their frequencies plotted as a true positive (TP) prediction rate in each Z-score bin (see coloured but not yellow bars in Figure 4).

The same process of TP estimation was then repeated with ten randomized versions built up from each of the four functionally related clusters datasets. The average frequency of random pairs found in each Z-score bin was taken as the false positive (FP) rate (see yellow bars in Figure 4). Precision was then calculated from the TP and FP rates with the formula: TP / (TP + FP) (see the declining black lines in Figure 4).

Within all four datasets analysed, it can be seen that higher Zs correlate with higher frequencies of TP predictions and with lower rates of FP predictions (see Figure 4). The Phylo-Tuner method's precision in distinguishing TP from FP cases is around 90% in the highest Z-score bin (Zs ≤ −3.5), drops to around 80% in the lower Z-score bin range (between −3.5 and −3.0), and in all cases gradually decreases to the random proportion of 50% when the Z-score values are in the range −1.0 to −0.5.

If the Phylo-Tuner method's precision rates are assessed over the entire Z-score distributions of all compared cluster pairs, we see that almost 90% of the 2,344 pairs (formed by 352 different clusters) with Zs ≤ −3.5 (2,109 pairs) are true functionally related clusters. If the slightly lower Z-score boundary is considered (Zs ≤ −3.0), around 80% of the 5,255 pairs (736 different clusters) found below this Z-score value (4,204 clusters pairs) would be expected to correspond to TP predictions. Files containing predicted pairs with Zs ≤ −3.0, and information to generate the profile matrix and perform validation of the Phylo-Tuner method using GO, are provided on the ftp site ftp://ftp.biochem.ucl.ac.uk/ pub/ gene3d_data/ CURRENT_RELEASE/ PHYLOTUNER/. (We also provide files with the new prediction results using an updated profile matrix with 26 eukaryotic species at the same ftp site).

Also interestingly, for all cluster pairs with Zs ≤ −3.0, only 3% correspond to instances of domains that frequently co-occur in the same proteins. This indicates that the Phylo-Tuner is able to identify a strong co-evolutionary signal between domains that is not simply due to their fusion in the same gene.

These results clearly indicate that multigene eukaryotic protein clusters with similar phylogenetic profiles tend to be functionally related and confirm the evolutionary theory behind the Phylo-Tuner method, which holds that functionally linked eukaryotic multigene families have co-evolved and varied their numbers of gene copies in a codependent fashion throughout the speciation process.

The specificity and sensitivity of the method were not calculated due to the difficulty in obtaining reliable datasets for true negative and false negative predictions. It is highly probable that two functionally related protein families divide into clusters at different levels that retain some functionally related intersections from the parental families, but in many cases without similar profiles. Therefore, whilst it is expected that highly significant profile similarities would indicate highly functionally related protein clusters, it is not necessarily true that functionally related clusters always present profiles with high similarity. This lack of transitivity makes it very difficult to establish with real confidence the false negative predictions produced by the method, and therefore how the method's sensitivity rate varies with respect to the profile similarity Zs.

Similarly, we do not have a reliable dataset of functionally unrelated protein clusters (true negative predictions) to estimate the Phylo-Tuner method's specificity, since the GO annotation of the domain sequences in our dataset is clearly incomplete (∼50%) and restricted to the human genome; therefore, many functional relationships have probably been missed from the functionally related clusters' datasets.

### Comparing the Performance of Phylo-Tuner with the Presence–Absence Phylogenetic Profiles

**Figure 5:**
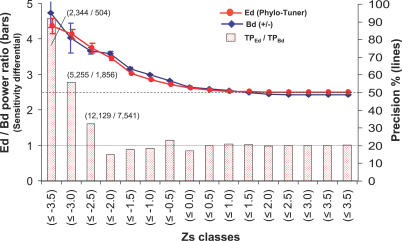
Comparison of Ed versus Bd Methods The power or sensitivity differential between the methods is measured by the (Ed's TP)/(Bd's TP) prediction ratios (bars; left-hand *y*-axis) for every Zs class (*x*-axis). The number of Ed and Bd TPs are indicated for the three highest Zs levels (figures in brackets). Also shown is the precision percentage distribution (right-hand *y*-axis) for the Bd (blue line) and Ed (red lines methods) by Zs value classes. Standard deviation (vertical lines), the random precision 50% value (dashed line), and the neutral sensitivity differential ratio of value 1 (dotted line) are also indicated. In the traditional language of statistical hypothesis testing, the sensitivity of a test is called the statistical power of the test. A more sensitive test will have fewer type II errors. The type II error is the “false negative” error, or the probability of rejecting a TP prediction. Since Ed and Bd statistics are run on the same matrix (sample), the total number of TP pairs is the same for both methods. Considering this premise, and that both methods show the same precision at all Zs significance levels, the ratios (Ed sensitivity)/(Bd sensitivity) and (Ed power/ Bd power) are equal to the ratio (Ed TP rate)/(Bd TP rate) for every Zs bin.

Ed and binary (present–absent) or bit distances (Bds) were calculated for all cluster profile pairs on the same matrix of 3,721 protein clusters and assessed with the same procedure as described in the former section (for the Bd calculations all cluster profiles were converted into the presence–absence type). Precision and statistical power differential ratios (equivalent to sensitivity differential ratios) were calculated for both methods using their respective TP and FP rates for each Zs bin (see Figure 5).

Both methods show the same precision at all levels of significance (see blue and red lines in Figure 5). However, Ed statistics show it to be more powerful than the Bd approach when the Zs values increase toward the highest significant levels (see bars in Figure 5). Ed predicts 4.6-fold more TPs than Bd for Zs ≤ −3.5 (both with 90% precision); almost 3-fold more sensitive for Zs ≤ −3.0 (80% precision); and 1.6-fold more sensitive for Zs ≤ −2.5 (>70% of precision). Additionally, the Ed values show no correlation with the Bd values calculated for the same sample of significant pairs with Zs ≤ −3.0 (see Figure S2). This lack of correlation indicates that the Phylo-Tuner method retrieves independent and additional predictions by exploiting the profiles' Ro information content.

### Example Predictions by Phylo-Tuner

Many of our predictions can be considered “novel” since it is generally difficult to find examples of functional association predictions that have clear supporting evidence in the literature. As examples of novel functional relationship predictions, we have selected ten pairs of clusters with high statistical significance that we consider to be very promising targets for experimental validation (see Table S1). Detailed functional analysis of the literature revealed particular links between some genes in the majority of the ten selected pairs (see Text S1), suggesting that the functional relationships between these predicted pairs merits further experimental validation. To enable experimental validation of our method by the scientific community, the Phylo-Tuner profiles and source code presented in this work will be made freely available from the same Gene3D ftp site given in the section Assessment of Profile Similarity and Predicting Functionally Related Clusters above.

Two pairs of protein domain clusters with significant Zs (Zs ≤ −3.0)—and a clear functional relationship in the literature—were selected for more detailed comparison between our method and a standard presence–absence profile analysis (see Table 1). These two pairs were selected as examples because of extreme differences in gene family representations in the species (one pair was present in all organisms, and the other pair was present in only six species), and their different functional roles in the cell (one is involved in the cellular cytoskeleton, and the other in the regulation of cellular differentiation).

**Table 1:**
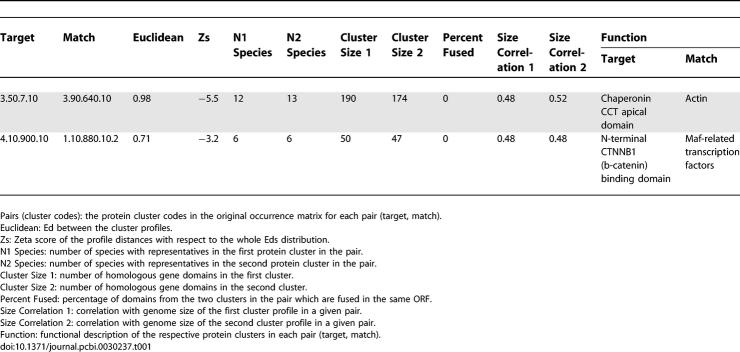
Profile Comparison Scores and Statistics for Two Example Pairs

To test how robust the Phylo-Tuner method is to noise, we decided to add back into the occurrence matrix additional information from profiles with representations from less than six species (e.g., four and five species) that we had previously removed from our analysis to see whether we could still detect a signal. Profile comparison and analysis of the cluster pairs in our example were therefore performed using an extended version of the original matrix. This extended matrix was made by adding to the 3,721 clusters' profiles of the original matrix (see Materials and Methods section) the 20,954 clusters' profiles with representation in four or more species, and without the application of the 0.1 ≤ Ro ≤ 0.4 information boundaries threshold.

### The CCT Chaperone and Actin-Like Families

The first pair of clusters examined comprises different CATH domains at the superfamily level: 3.50.7.10 and 3.90.640.10. Genes from these clusters are distributed throughout the entire sample of organisms (see Table 1). Cluster 1, 3.50.7.10, corresponds to the apical domain of the eukaryotic CCT chaperonin subunit involved in substrate binding, whilst the 3.90.640.10 domain of cluster 2 constitutes the Actin-like protein family involved, amongst other functions, in cytoskeleton formation and protein folding. The CCT protein is the eukaryotic relative of the better-characterised prokaryotic chaperonin GroEL, sharing the same general monomer architecture of three domains: an equatorial domain that carries ATPase activity (cluster code: 1.10.560.10), an intermediate domain (3.30.260.10), and an apical domain, involved in substrate binding (3.50.7.10) [16]. In contrast to GroEL, the CCT chaperonin shows a more specific functional role dedicated to the folding of the cytoskeletal proteins actin and tubulin, and collaborating with the Hsp70 actin-like protein in the cytosolic chaperone network [17].

**Figure 6:**
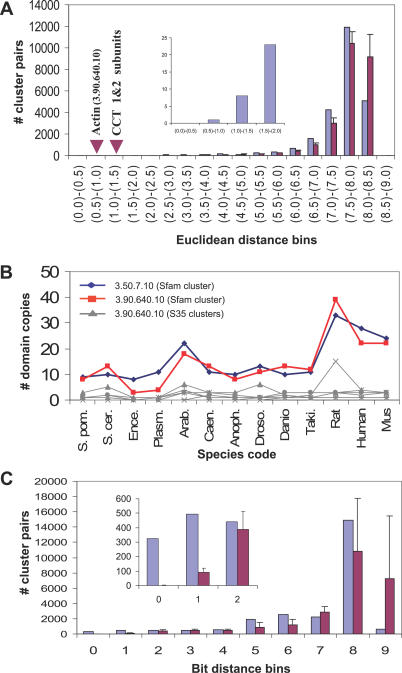
Profile Comparison of the 3.50.7.10 Domain Cluster against the Rest of Profiles in the Extended Matrix (See the Section, Example Predictions by Phylo-Tuner) (A) Number of cluster pairs (*y*-axis) in different Ed bins (*x*-axis) for the 3.50.7.10 cluster comparisons (blue bars). In addition, the average number of pairs for comparisons involving five randomisations of this cluster profile are also shown together with standard deviations (red bars and vertical black lines); the location of the closest related cluster, Actin-like (3.90.640.10), is indicated with a red arrow together with a closeup of the left-hand extreme of the distribution (upper smaller graph). (B) Number of domain copies (*y*-axis) for the 3.50.7.10 cluster (thick blue line), the cluster profile with the closest Ed (3.90.640.10, thick red line). Also shown is the number of domain copies in each of the S35 subclusters of the Actin 3.90.640.10 cluster. Further subdivisions were not shown to maintain clarity. (C) Number of cluster pairs (*y*-axis) in different Bd bins (for presence–absence profile comparison) (*x*-axis) for the 3.50.7.10 cluster comparisons (blue bars) and the average number of cluster pairs in each bin, for five randomised models of the same cluster, together with their standard deviations (red bars and vertical black lines).

In the Ed distribution of the CCT apical domain cluster (3.50.7.10) compared against the other 24,675 cluster profiles (see Figure 6A), the Actin cluster (3.90.640.10) shows the closest distance (Ed = 0.98, Zs = −5.5; see 0.5–1.0 bin in Figure 6A, and Table 1) followed next by nine other protein clusters. Amongst these nine protein clusters are found the CCT equatorial domain (cluster code: 1.10.560.10) and the intermediate domain (3.30.260.10) (see 1.0–1.5 bin in Figure 6A).

Profile comparison of the CCT and Actin-like clusters at different identity levels show that only when the actin-like proteins are clustered at the superfamily level does its profile give the closest match (shortest Ed) with the CCT apical domain superfamily profile and vice versa (see Figure 6B and Figure S3A). Therefore, without any prior assumptions in setting similarity thresholds for recognising and clustering orthologues, the Phylo-Tuner method is able to distinguish statistically significant co-evolution signals from different identity levels in the CCT and Actin-like families.

When all the cluster profiles in the profiles matrix are converted into the presence–absence type profile and their distances to the 3.50.7.10 profile are calculated in bits (as is typically performed for this type of analysis, see [3], also Figure 6C), more than 300 different protein clusters show short distances of 0 bits to the CCT terminal domain (3.50.7.10) cluster profile (see number of clusters in the 0 class bin in Figure 6C). The increase in the number of matches, and therefore in the statistical uncertainty, indicates that the use of occurrence information (i.e., copy variation throughout the species) provides an additional and independent approach (see Figure S3B) that significantly increases the precision and sensitivity for co-evolution signal detection.

### A beta-Catenin Binding Domain and the SMAF-1 Transcription Factor Family

The second example pair comprises clusters 4.10.900.10 and 1.10.880.10.2. The protein clusters in this pair show a more specific distribution being present in only six different species (see Table 1). The first corresponds to the N-terminal CTNNB1 binding domain, which appears to bind the armadillo repeat of CTNNB1 (beta-catenin) forming a stable complex. Beta-catenin is involved in the signalling stream of the Wnt regulatory pathway. The canonical Wnt signalling pathway regulates decisions in embryonic development through body axis specification and morphogenic signalling and its malfunctioning can cause some diseases, such as cancer [18,19].

The 1.10.880.10.2 cluster represents a family of transcription factors important in the regulation of embryonic development and cell differentiation, including oncogenic proteins [20]. Apart from the similar functional role of these two clusters described above, there is additional evidence that suggest a close functional relationship between relatives of these two protein families [21].

**Figure 7:**
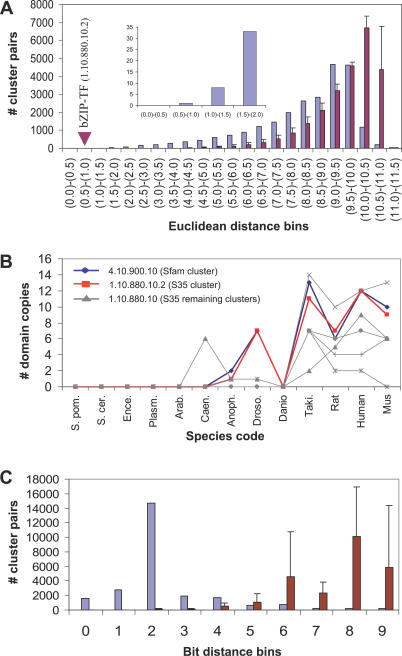
Profile Comparison of the 4.10.900.10 Cluster against the Rest of the Profiles in the Extended Matrix (See the Section, Prediction Examples by Phylo-Tuner) (A) Number of pairs (*y*-axis) in each Ed bin (*x*-axis) for the 4.10.900.10 cluster comparisons (blue bars) and the average of five randomised models together with their standard deviations (red bars and vertical black lines); the location of the closest profile for bZip transcription factor (1.10.880.10.2) is indicated with a red arrow, and a closeup of the extreme left-hand side of the distribution (upper smaller graph) is shown. (B) Number of domain copies (*y*-axis) for the 4.10.900.10 cluster (thick blue line). The cluster profile with the closet Ed (1.10.880.10.2, thick red line) is also shown together with the number of domain copies in other subclusters at the same sequence identity level (S35). (C) Number of cluster pairs (*y*-axis) in different Bd bins (for presence/absence profile comparison) (*x*-axis) for the 4.10.900.10 cluster comparisons (blue bars) and the average number of cluster pairs in each bin, for five randomised models together with their standard deviation (red bars and vertical black lines, respectively).

The Ed distribution of the 4.10.900.10 cluster profile, in comparison to the rest of profiles in the extended occurrence matrix, shows the 1.10.880.10.2 cluster as the most significantly close cluster (Ed = 0.71, Zs = −3.2; see 0.5–1.0 bin in Figure 7A, and Table 1), indicating a high probability of functional relationship and co-evolution amongst these two multigene families. As observed in the former example for the CCT and Actin clusters, the profiles comparison of the 4.10.900.10 and the 1.10.880.10.2 clusters at different identity levels show again that the Phylo-Tuner method is distinguishing statistically significant co-evolution signals at specific identity levels (see Figure 7B and Figure S4A).

As with the previous example, Bds calculated for the presence–absence version of the occurrence matrix again show no correlation with the Eds calculated on the same sample (see Figure S4B). Furthermore, the use of presence–absence information alone dramatically increases the uncertainty regarding putative functionally related partners for the 4.10.900.10 cluster. Bd distribution of protein clusters shows that almost 2,000 different profiles are significantly close to the 4.10.900.10 cluster profile (see 0 bin in Figure 7C). The more specific functional relationship with the 1.10.880.10.2 protein cluster is only separated from the other 2,000 clusters when the information on domain number occurrence is used, as in the Phylo-Tuner method, to increase the predictive power.

### Conclusions and Future Directions

Eukaryotic protein cluster profiles are poorer in R+/− and richer in Ro information content due to bigger average family sizes and wider gene-copy variation through species than for prokaryotes (Figure 2A–2C). We have found that by exploiting the Ro information resource in eukaryotic profiles, the Phylo-Tuner method is able to find co-evolutionary signals amongst functionally related multigene families (see Figure 4) that could not have been predicted by the standard presence–absence profile methods (see Figures 5–7, and Figure S2).

We have demonstrated that two protein clusters will “tune” with a significant correlation signal when their profiles are compared at the sequence similarity levels that capture their functional relationship (see Figures 6A, 6B, 7A, and 7B; and Figures S3A and S4A). Therefore, subdividing protein families into discrete sequence identity levels is a novel implementation of phylogenetic occurrence matrices with three main advantages. Firstly, it takes into account the wide variation in gene copy number observed in eukaryotes. Secondly, it encapsulates a more realistic model of the variation that uneven natural selection presser produces on different protein families and organisms. Thirdly, it does not require the initial application of rigid similarity E-value boundaries or complex protocols for orthology assignment. This last advantage is especially useful since the bigger average size of eukaryotic multigenes families makes them more prone to orthology mis-assignment than in prokaryotes.

Regarding protein families clustered at different sequence identity levels, it would clearly also be possible to use other approaches to clustering, for instance based on discrete subdivisions of phylogenetic trees. That is, selecting protein clusters associated with different nodes within each family's phylogenetic tree and generating the occurrence profiles from these tree-based subdivisions.

Protein cluster profiles in prokaryotes typically show an average representation of one copy per species (see Figure 2C). This feature of gene copy distributions in prokaryotes allows the prediction of specific functional relationships between proteins using “presence–absence” profiles and standard profiling methods. However, protein clusters in eukaryotic genomes are frequently represented by more than one gene copy per organism (see Figures 2C, 6B, and 7B). Therefore, whilst the Phylo-Tuner method can clearly detect functional relationships between eukaryotic protein clusters, because there is generally more than one gene copy per species, it cannot identify the specific orthologues involved in the functional association amongst all the homologous genes within the same cluster.

To illustrate this point by referring to one of the examples described above, the pair of protein clusters comprising the N-terminal beta-catenin binding and the Maf-SKN-1-like transcription factor domains (see second row in Table 1) are each represented by 12 paralogous genes in the human genome (see Figure 7B). Although, the Phylo-Tuner method significantly reduces the uncertainty in selecting two specific profiles of functionally related clusters amongst thousands of possibilities, some uncertainty still remains with respect to the specific coupling of the 12 pairs of interacting genes from these human protein clusters.

Therefore, to reduce the remaining uncertainty associated with the identification of the orthologous genes involved in the specific functional association for a particular organism, it would clearly be valuable to combine the Phylo-Tuner method with other prediction methods, such as those exploiting the correlation of phylogenetic tree topologies between superfamilies, i.e., [22,23]. Since, tree comparison algorithms are often overburdened by the combinatorial nature of the problem and by all the comparisons between functionally unrelated clusters, the prior application of the Phylo-Tuner method could significantly reduce the search space and improve the performance of these algorithms.

## Materials and Methods

### Domain annotation of eukaryotic and prokaryotic genomes.

ORFs from 13 complete eukaryotic genomes were structurally annotated by scanning the protein sequences against representative Hidden Markov models (HMMs) from the CATH domain structure database [24]. The 13 annotated eukaryotic species are: Encephalitozoon cuniculi (Fungus), Schizosaccharomyces pombe (Fungus), Saccharomyces cerevisiae (Fungus), Danio rerio (Fish), Takifugu rubripes (Fish), Plasmodium falciparum (Protozoan), Anopheles gambiae (Insect), Drosophila melanogaster (Insect), Arabidopsis thaliana (Plant), Caenorhabditis elegans (Nematoda), Rattus norvegicus (Mammal), Homo sapiens (Mammal), and Mus musculus (Mammal). The structural annotation data is available from release 3 of the Gene3D database [25]. 192,655 domain sequences were annotated in the eukaryotic sample in Gene3D, with an average coverage of 36% (S.D. = 13%) of the genes for the 13 complete genomes.

For generating a comparable prokaryotic dataset, the same domain annotation procedure was performed on 106 complete prokaryotic genomes, made up of 16 Archaeal and 90 Eubacterial species (see Table S2). 276,098 domain sequences were annotated in the eukaryotic sample in Gene3D, with an average coverage of 45% (S.D. = 6%) of the genes for the 106 complete genomes sample.

### Clustering protein domain families into sequence similarity levels.

Sequences are assigned to CATH superfamilies through the identification of significant matches to the CATH HMM library. These hits are then resolved to produce a non-overlapping set of domain assignments. These superfamilies form the root of the clusters. Every domain sequence in the family is then BLASTed [7] against each other to produce a similarity matrix based on sequence identity. This matrix is then used to produce the clusters at 30%, 35%, 40%, 50%, 60%, 70%, 80%, 90%, 95%, and 100% (see Table S3) by using multi-linkage clustering—whereby every sequence in a subcluster will exhibit at least that degree of sequence identity to each other [25].

### Building the Gene3D phylogenetic occurrence profile matrices.

Occurrence profiles were calculated for all the protein domain clusters (superfamilies and subclusters) in the eukaryotic and prokaryotic samples at different identity levels (see Figure 1). Occurrence profiles were derived for all the clusters from the number of domain copies observed in each species (Figure 1).

Sometimes the domain content of clusters did not change when subsequent levels of identity percentage were applied (e.g., compare s30 (A) and s35 (A) levels in Figure 1). Therefore, subclusters having the same domain content and, hence, occurrence profile as their parental clusters were detected and removed.

### Measuring the similarity of occurrence profiles.

In contrast to the prokaryotic sample, the genome sizes of the eukaryotic sample are not homogeneously distributed, but instead form three heterogeneous groups (see Figure S1A and S1B). This heterogeneous distribution introduces a significant bias if the similarity of a pair of occurrence profiles is calculated using correlation indexes such as Pearson and increases the likelihood of a spuriously high correlation value. To avoid this problem, Ed was selected for measuring the distance between pairs of profiles. Ed is sensitive to scaling and differences in average domain numbers in protein clusters, whereas a correlation index is not [26].

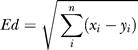



When the Ed of the profile pairs are plotted against the mean of their domain number averages for the eukaryotic and prokaryotic samples (see Figure S5A and S5C), it can be seen that the data are heteroscedastic, so that error variance in the Ed values is proportional to the domain number averages. When both variables (Ed and the mean of profile averages) are transformed with logarithmic functions, a linear relationship is observed between these variables (see Figure S5B and S5D).

Therefore, because the distance error is proportional to the profiles' average size, to normalise the error and make it comparable for all profile pairs with different domain number averages, the Ed was divided by the mean of the cluster sizes (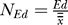 , where N_Ed_ and E_d_ are the normalised and original Ed, respectively, and 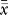 is the mean of the sizes of the cluster pair). This normalised Euclidean value was used to measure the distances in the all-against-all comparison of profiles.

If a cluster was a subset of another cluster, then distance calculations were not carried out. This is because such profiles are likely to show similarity simply because the former contains several of the elements of the latter and not for any evolutionary or functional reason.

We also studied the statistical impact of homology on the performance of Phylo-Tuner, arising from the profile comparisons of separate subclusters in the same superfamily. Homologous pairs were found to count for only 6% of all pair comparisons, and their inclusion does not significantly affect the overall performance of the Phylo-Tuner method (see Figures S6 and S7). However, when homologous clusters' profiles show significant similarity, it may be indicative of true co-evolutionary signals, and therefore we have included them within our analyses.

### Using Z scores to assess the significance of associations.

Each profile has a collection of Ed values resulting from its comparison with the other profiles. Generally, the distance values for all the clusters showed a normal distribution. Therefore, the average (*x*) and standard deviation (*s*) were calculated for each collection of distances from each profile and Zs were calculated for each distance value (N_Ed_) within each distribution (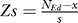 ). Zs is a normalised parameter that can be used for comparing different pairs of clusters and their distributions.

### Examining the correlation between profiles and genome size.

Pearson's method was used to measure the correlation coefficients between the similarity of occurrence profiles and genome sizes.

### Measuring information content: Presence–absence information (R+/−) and occurrence information (Ro).

It is important to develop methods to measure the information content of the profiles, since it has been demonstrated that profiles with low information content introduce noise into the calculation of the correlation between similarity in profiles and the prediction of functional relationships [3]. In our model, we define two kinds of information: one is related to the presence–absence pattern of the protein clusters throughout the different species and is similar to that used by other groups, i.e., [3]. This presence–absence information will be referred to as R*+/−*. The other information measure is related to the variation in gene copy number throughout the species in the profiles and this will be referred to as Ro or occurrence information.

R+/*−* is calculated using the Shannon formula R+/*−* = Hb − Ha, where Hb and Ha are entropy measures of the information held by the receptor before (Hb) and after (Ha) it receives the profile message. Therefore R+/*−* is a measure of the reduction in uncertainty of the receptor once the message is received (in this case the message is the distribution of +/*−* in each profile). It can be assumed that the receptor previously knew the number of species in the reference sample, and how many presences (+) and absences (*−*) there were in the profile, but has no knowledge of the distribution of these +/*−* across the different species. In this case, the receptor entropy before receiving the message will be 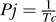 , where Pj is the probability to find any single distribution with a determined number of + and *−* in a profile *j* amongst the total number of all possible distributions of +/*−* in the same profile j. The total number of possible combinations (Tc) is calculated with the formula 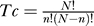 , where *N* is the number of different species (positions) in the profile and *n* is the number of elements to combine (e.g., + or *−*). Therefore, 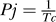 since the receptor can expect to receive the message in any of the possible combinations. Once the receptor receives the message, the entropy Ha goes to zero.

Ro is calculated using an interpretation of the Shannon formula known as the SBI, Shannon Biodiversity Index (also known as Shannon-Wiener Index) as a measure of entropy. In the original formula: 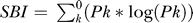 , where *k* is the number of different species in a given ecosystem, and *Pk* is the proportion of individuals in the species *k* amongst all individuals in all the species in the same ecosystem. In our interpretation, *k* is the number of eukaryotic genomes (species) in the profile, and *Pk* is the proportion of domain number copies in each genome *k* amongst all domain copies for a particular superfamily in all genomes. Ro measures the uncertainty reduction variation produced in the receptor before and after receiving the profile occurrence message: Ro = Hb − Ha. In the Ro estimation, it is assumed that the receptor knows how many species hold gene domain cluster representatives, and the total number of copies in the profile, but has no knowledge of how the copy numbers vary throughout the species. The receptor expects that any domain has the same probability to occur in any of the genomes, implying equally probable distribution of domain copies across genomes, then 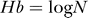 , where N is the number of species that hold domain copies. Once the receptor receives the profile, the proportion of domain copies in each species will be known *k* (*Pk*), and the new information reduces its uncertainty 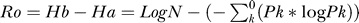 .

### Construction of null models.

Two random models were built for comparison: (1) genome shuffling (GS) and (2) profile shuffling (PS). These two random models were compared against the occurrence profiles matrix for the real protein clusters to estimate the statistical significance of the similarity distances between cluster profiles.

(1) Genome shuffling:

*Purpose.* To estimate of the effect that genome size has on the Eds between profiles.

*Method.* All known domains for the 120 genomes were put into a single array and shuffled randomly. Then the array was split according to the known genome sizes to create a set of pseudo-genomes. These were used to construct the family profiles as before.

*Outcome.* These pseudo-genomes have the same sizes as the real set but have a randomly chosen set of domains allowing us to estimate the effect that genome size has on correlation values.

(2) Profile shuffling:

*Purpose.* To estimate the effect that domain family size has on the Eds between profiles.

*Method.* Each S100 profile was shuffled, so that the values were assigned to a genome at random. This was done independently for each profile at the S100 level. Then each profile at the higher levels was regenerated using these values.

*Outcome.* This effectively makes every genome approximately the same size and with a generic domain set thus allowing us to estimate the effect of domain family size on correlation values.

### Selection of profiles with a statistically significant species representation.

There are no absolute criteria to choose an optimum minimum number of species for profile selection. If the threshold is very restrictive, there is likely to be an increase in precision (i.e., less FP predictions) and a decrease in sensitivity (more false negative predictions), and vice-versa. Analogous microarray data analyses use Ed to measure gene expression profile similarity, and in this application evidence suggests that five biological cases (species) is the minimum to analyse microarray data with some guarantee of statistical robustness; however, this minimum is not necessarily an optimum [27].

We decided to use six species with positive gene presences as the minimum number for selecting profiles since it is above the five species mark, which clearly increases the precision of the approach (from 85% to 95%; see Figure S8). Furthermore, since in our species dataset there are no more than three species in any phyla, any profile with gene representation in six organisms has a guaranteed species representation in at least two out of the six possible phyla (mammals, fish, insect, nematode, fungi, plant). This decreases the possible error arising from the comparison of profiles with monophyletic origin [5]. Applying this threshold, a matrix with 10,005 protein domain cluster profiles, with representation in six or more species, was selected for the eukaryotic sample, and a matrix with 28,080 cluster profiles was selected for the prokaryotic sample.

### Measuring GO semantic similarity.

To validate the method, we chose to analyse our predictions with the Gene Ontology (GO) database [28], which allowed us to implement a consistent measure of the functional relationships between protein clusters.

A semantic similarity (SS) score was calculated for each pair of GO terms in an all-against-all comparison of GO terms annotated in all human domain sequences in the Gene3D database. This was done using an implementation of the method described in [29], an approach which measures the “information content” of GO terms based on their relative frequency of appearance in a given context (in this case the whole pool of protein sequences in the human genome). For example, thousands of sequences are annotated with the term “kinase activity” (GO:0016301) in human, while the term “recombinase activity” (GO:0000150) only appears about six times. Therefore, the probability that two sequences will share the “recombinase activity” annotation by chance is much less likely than if they shared the “kinase activity” one. Furthermore, the information content of the “recombinase activity” term is much higher than the “kinase activity”, since in contrast to the “recombinase activity”, the “kinase activity” can be linked to hundreds of different biological processes and cellular components in human.

The frequencies of all the GO terms in each of the three GO categories were calculated using the human domain sequences GO annotations file and the hierarchical parent/child relationship information obtained from the OBO flat file downloaded from the GO database (30 December 2005 release). These frequencies were converted into probabilities by dividing them by the maximum frequency value in each independent GO category. An SS was then calculated for each pair of GO terms in the all-against-all comparison by taking the minimum probability (pms) amongst the probabilities of all the parental GO terms shared by every possible pair (c1 and c2). SS values were calculated using the Resnik formula (1990) as explained in [29].

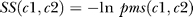



### Validation of associated profiles with GO annotations and SS scores.

The identification of functionally related cluster pairs was performed in four stages. 1) Calculation of the SS scores of all pairs of GO terms annotated in the complete set of human domain sequences present in Gene3D and estimation of statistically significant thresholds for the selection of highly informative SS scores. 2) Selection of functionally related domain sequence pairs by using highly informative GO SS scores. 3) Validation of functionally related protein clusters based on the functionally related domain pairs identified in 2) (details given below). 4) Assessment of significance by randomisation of the datasets to enable estimation of the FP rates. (Note: We attempted the above calculations with all genomes in the complete eukaryotic sample and not just human, but the huge CPU time required made the analysis impractical, and thus all calculations were subsequently performed just using the human subset.)

**Figure 8:**
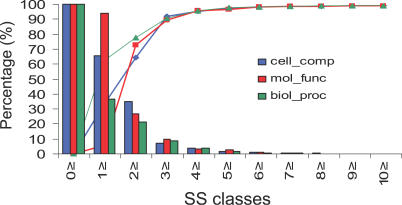
Analysis of the SS of GO Terms in the Human Genome for the Three GO Functional Classes Percentage of SS scores for GO pairs (bars) in each SS bin (*x*-axis). Statistical confidence (lines), expressed as a percentage, for distinguishing TP SS relationships from random is also shown for different SS score bins (*x*-axis) and for different GO functional groups—cellular components (blue); molecular function (red); biological processes (green). To assess the statistical confidence, random GO pair frequencies are calculated in each SS bin for the human genome (P_error_), and thus the statistical confidence can be then expressed (as a percentage) as the complementary probability: (1 − P_error_)*100.

*Step 1.* 56% of the 32,757 human CATH protein domains found in Gene3D, (18,253 domains) were found to have functional annotation in at least one of the three categories of the GO database: molecular function; biological process; and cellular component. SS scores were calculated for all pairs of GO terms, and the complete SS value distributions were plotted for the three different GO categories (see Figure 8). These SS distributions were used to establish SS value boundaries for selecting GO pairs with high SS scores and therefore with highly informative functional relationships.

1,000 GO pairs with SS values ≥5.0 were selected for the cellular component category, since this highly informative set accounted for only 1.9% of all pairs in the distribution (precision = 98.1%; see Figure 8). A set of 4,391 GO pairs (0.85%, precision = 99.15%; see Figure 8) with an SS score ≥6.0 were selected for the molecular function category, and 4,453 GO pairs (0.65%; precision = 99.35; see Figure 8) with SS scores ≥6.0 were selected for the biological process category. These data will be referred as the highly informative GO pair sets.

*Step 2.* A dataset of domain pairs predicted to have a significant functional relationship, was generated by selecting domain pairs sharing any of the highly informative GO pairs identified in Step 1 above. This gave 135,936 highly informative domain pairs for the cellular component category, 176,606 domain pairs for molecular function, and 243,383 for biological process. These data will be referred to as the highly informative domain pairs.

*Step 3.* To identify functionally related protein clusters, all the domain sequences from a given cluster were compared against all the domain sequences from another, and clusters pairs sharing at least one highly informative domain sequence pair were selected. To increase statistical confidence in these selected, functionally related clusters, the following thresholds were applied: a) all protein clusters compared had to have at least two human sequences annotated in GO; b) if each of the clusters compared had only two human sequences annotated in GO, they had to possess at least two highly informative domain pairs out of the four possible domain pair combinations (2 × 2); and c) any remaining cluster pairs were selected if at least 25% of the domain pairs, out of all possible combinations, were highly informative.

Using these selection criteria, three datasets of functionally related clusters were identified: 22,968 functionally related cluster pairs comprising 1,175 different domain clusters were selected for the cellular component set; 25,032 functionally related clusters pairs comprising 1,951 different domain clusters for the molecular function set; and 55,912 cluster pairs comprising 2,156 different domain clusters for the biological process set. An extra set of functionally related clusters was obtained by combining all data in these three sets. Redundant cluster pairs shared by the different sets were only included once in this combined set. In general, the three different sets of functionally related clusters showed small intersections amongst their data. 31,604 cluster pairs were finally combined, after removing redundancy between the sets (4,652 pairs or 12.8% of total pairs; see Table S4).

*Step 4.* To estimate the FP rate expected at random in each different set of functionally related clusters, every cluster was randomly paired with another cluster ten times and the average Z-score distributions recalculated.

## Supporting Information

Figure S1 Species Ordered by Their Genome Sizes in the Eukaryotic and Prokaryotic Samples Species (*x*-axis) ordered by their genome sizes (*y*-axis) in the eukaryotic a) and prokaryotic b) samples. Genome size is measured as the number of sequence domains found in the Gene3D database. Whilst the prokaryotic sample shows an almost continuous representation of genome size values, the eukaryotic sample shows a heterogeneous distribution with at least three different groups: groups 1, 2, and 3 in the a) plot.
(53 KB PPT)

Figure S2 Comparison of Euclidean and Bit Values Comparison of Ed and Bd values. Comparison for the sample of significant predictions (Zs ≤ −3.0) provided by the Phylo-Tuner method.
(A) Ed (*y*-axis) versus Bd (*x*-axis), and (B) Zs values based on Ed (*y*-axis) and Bd (*x*-axis) distributions. Trend line equations, R-squared values, and correlation coefficients are also indicated.
(712 KB PPT)

Figure S3 Profile Comparison of the 3.50.7.10 Domain Cluster against the Rest of Profiles in the Extended Matrix Profile comparison of the 3.50.7.10 domain cluster against the rest of profiles in the extended matrix (see Example Predictions by Phylo-Tuner section).
(A) Number of domain copies (*y*-axis) for the 3.90.640.10 cluster (thick blue line) is shown and for the cluster with the closest Ed (3.50.7.10—CCT—thick red line). The number of domain copies in the different subclusters (S35) of the 3.50.7.10 CCT cluster are also shown.
(B) Ed (*y*-axis) versus the corresponding Bd (*x*-axis) for the comparison of 3.50.7.10 cluster against all other clusters.
(339 KB PPT)

Figure S4 Profile Comparison for the 4.10.900.10 Cluster against the Rest of the Profiles in the Extended Matrix Profile comparison for the 4.10.900.10 cluster against the rest of the profiles in the extended matrix (see Example Predictions by Phylo-Tuner section).
(A) Number of domain copies (*y*-axis) for the 1.10.880.10.2 cluster (thick blue line), for the cluster with the closest Ed (4.10.900.10, thick red line). The number of domain copies in different subclusters (S35) of the 4.10.900.10 Nt b-catenin binding subunit cluster are also shown.
(B) Ed (*y*-axis) versus Bd (*x*-axis) for the comparison of 4.10.900.10 cluster against all other clusters.
(230 KB PPT)

Figure S5 Profile Similarity Score Analysis For eukaryotic (A) and (B) and prokaryotic (C) and (D) profiles.
(A,C) Eds for profile pairs (*y*-axis) versus the average sizes of the profiles (*x*-axis).
(B,D) Logarithm of the Eds for the profile pairs (*y*-axis) versus logarithm of the average profile sizes (*x*-axis).
(484 KB PPT)

Figure S6 Comparison of the Analysis with Homologous Pairs against the Analysis without Homologous Pairs The frequency distribution (left-hand *y*-axis) of TPs and FPs is plotted for the original analysis with homologous pairs (blue and light blue, respectively) and without (red and pink, respectively). The percent precision distribution (right-hand, *y*-axis) is shown for the analysis with homologous pairs (blue line) and without (red line) for all Zs value bins (*x*-axis for both distributions). Standard deviations are also indicated for the FP rates (vertical lines). Precision percentages were calculated based on the TP and FP frequencies for every Zs bin in the “no homologous pairs” sample, using for this new analysis the same profile matrix (with 3,721 protein clusters) and following the same procedure as described in the section Validation of Associated Profiles With GO Annotations and SS Scores (Material and Methods) and the section Assessment of Profile Similarity and Predicting Functionally Related Clusters (Results/Discussion). It can be seen in this plot that the TP rate in the “no homologous pairs” sample drops slightly from 0.041% to 0.035% in the highest Zs class (≤−3.5), compensated by a proportional decrease of the FP rate in the same Zs bin, giving virtually no variation in the precision ratio compared to the original analysis. For the remaining Zs bins, no significant differences are observed in the TP rate, the FP rate, or the precision values, demonstrating that no significant upward bias of the precision ratio is caused by the inclusion of homologous pairs in the analysis.
(60 KB PPT)

Figure S7 Pairwise Ed Distributions by Percentage Pairwise Ed (*x*-axis) distributions by percentage (*y*-axis).
(A) The homologous (blue line) and the nonhomologous (red line) pairs.
(B) Distributions for the clusters in the homologous 3.40.50.300 superfamily (blue line) against the rest of clusters in the matrix (red line). Using the same matrix of 3,721 protein clusters referenced above, the Ed distributions of homologous and nonhomologous pairs were calculated independently and compared between both sets (A). Homologous pairs count for only 6% of all pair comparisons, and these pairs therefore have a low statistical weight in the whole statistical analysis. From this comparison it can be seen that the homologous pairs show a very slight bias toward lower Eds. However, if the Ed distribution of homologous pairs from the superfamily 3.40.50.300 (a superfamily which is large enough for significant statistical comparison) is compared with the distance distribution for all the remaining 3.40.50.300 nonhomologous clusters (B), no significant difference is seen. Therefore, the likelihood of finding significant partners within or outside the superfamily are practically the same. These results indicate the possibility that homologous clusters could be co-evolving in a similar manner to nonhomologous pairs when a functional association between them is retained in evolution. For this reason, the co-evolution signal arising from the comparison of homologous profiles is retained within the Phylo-Tuner analysis.
(61 KB PPT)

Figure S8 Percentage of Profile Pairs in Each Ed Bin for Eukaryotic Profiles with Gene Representation in Six or More Organisms Percentage of profile pairs (*y*-axis) in each Ed bin (*x*-axis) for eukaryotic profiles with gene representation in six or more organisms (A) and in five or more organisms (B), for the real matrix (blue), the GS model (pink, GS), and for the PS model (yellow, PS).
(C) Analysis of the increase in precision obtained by having at least six species in the profile. The plot on the right hand of (C) shows, for the smallest Ed bin, the percentage of profile pairs from the real matrix as TPs (TP5), and the percentage of profile pairs from the random models as FPs (FPps5 and FPgs5). In the left-hand plot of (C), the same is shown but for the six species matrix.
(D) Precision values are estimated for the two different sources of FPs (PS and GS random models) and for the two different real matrices: five and six species.
(64 KB PPT)

Table S1 Ten Examples of Predicted Cluster Pairs with Novel Functional Relationships (36 KB DOC)

Table S2 The 106 Prokaryotic Species Used in the Analysis (118 KB DOC)

Table S3 Clustering of the 192,635 Domain Sequences (29 KB DOC)

Table S4 Number of Functional Clusters Pairs Selected in Each Functional Group Dataset (25 KB DOC)

Text S1 Detailed Bibliographic Analysis of the Ten Pairs Selected for Table S1
(52 KB DOC)

## Abbreviations

Bd: binary or bit distance
Ed: Euclidean distance
FP: false positive
GS: gene shuffling
HMM: hidden Markov model
ORF: open reading frame
PS: profile shuffling
SS: semantic similarity
TP: true positive
Zs: Z scores
