# Supplementary material for: Predicting Protein Function with Hierarchical Phylogenetic Profiles: The Gene3D Phylo-Tuner Method Applied to Eukaryotic Genomes
Source: PLoS Comput Biol. 2007 Nov 30;3(11):e237. doi: 10.1371/journal.pcbi.0030237 (PMC2098864; doi:10.1371/journal.pcbi.0030237)
Supplement: Figure S1 — Species (x-axis) ordered by their genome sizes (y-axis) in the eukaryotic a) and prokaryotic b) samples. Genome size is measured as the number of sequence domains found in the Gene3D database. Whilst the prokaryotic sample shows an almost continuous representation of genome size values, the eukaryotic sample shows a heterogeneous distribution with at least three different groups: groups 1, 2, and 3 in the a) plot. (53 KB PPT) [file pcbi.0030237.sg001.ppt]

## Slide 1
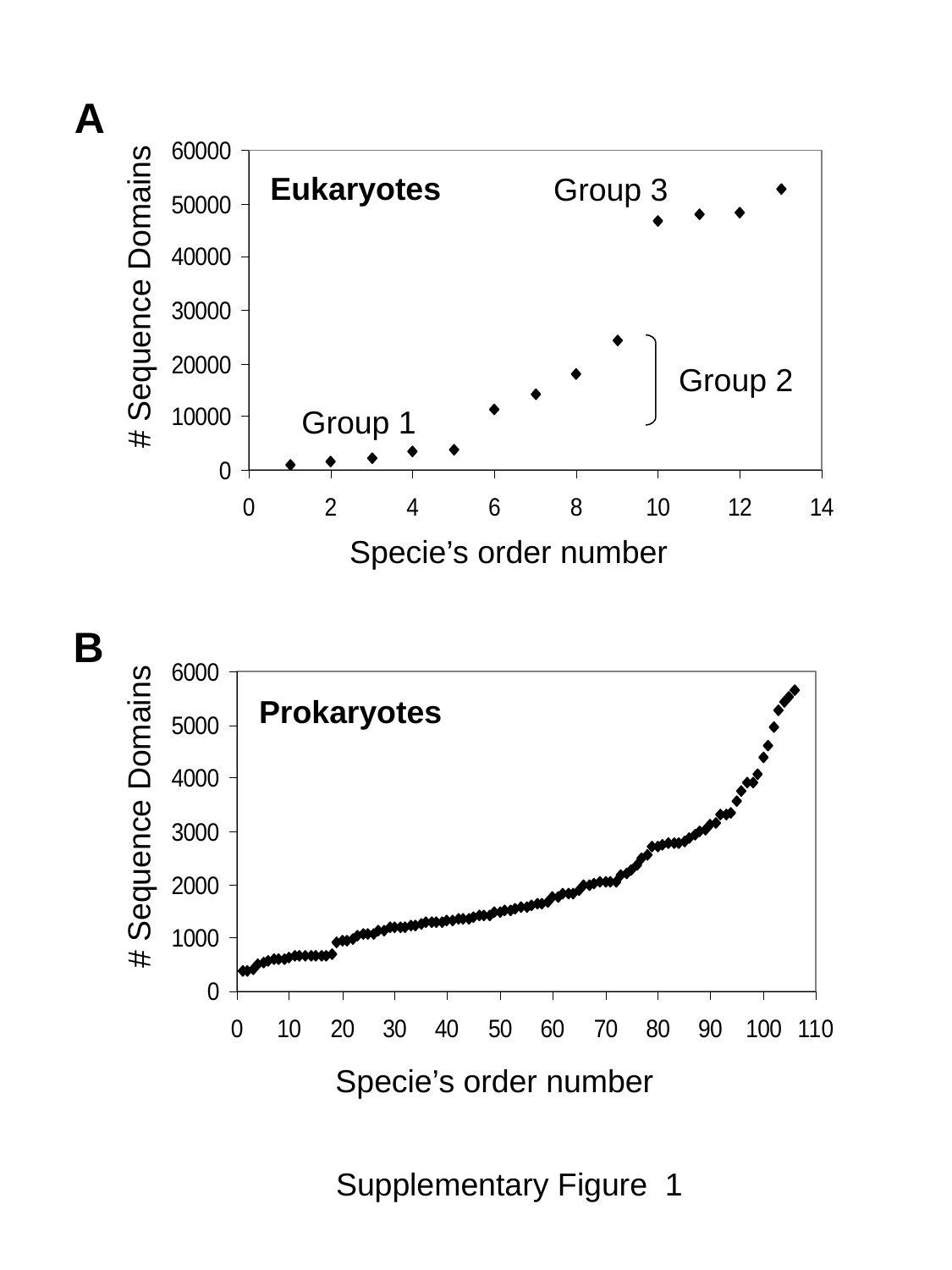

A
# Sequence Domains
Eukaryotes
Group 3
Group 2
Group 1
Specie’s order number
B
# Sequence Domains
Prokaryotes
Specie’s order number
Supplementary Figure 1
