# Supplementary material for: Predicting Protein Function with Hierarchical Phylogenetic Profiles: The Gene3D Phylo-Tuner Method Applied to Eukaryotic Genomes
Source: PLoS Comput Biol. 2007 Nov 30;3(11):e237. doi: 10.1371/journal.pcbi.0030237 (PMC2098864; doi:10.1371/journal.pcbi.0030237)
Supplement: Figure S2 — Comparison of Ed and Bd values. Comparison for the sample of significant predictions (Zs ≤ −3.0) provided by the Phylo-Tuner method. (A) Ed (y-axis) versus Bd (x-axis), and (B) Zs values based on Ed (y-axis) and Bd (x-axis) distributions. Trend line equations, R-squared values, and correlation coefficients are also indicated. (712 KB PPT) [file pcbi.0030237.sg002.ppt]

## Slide 1
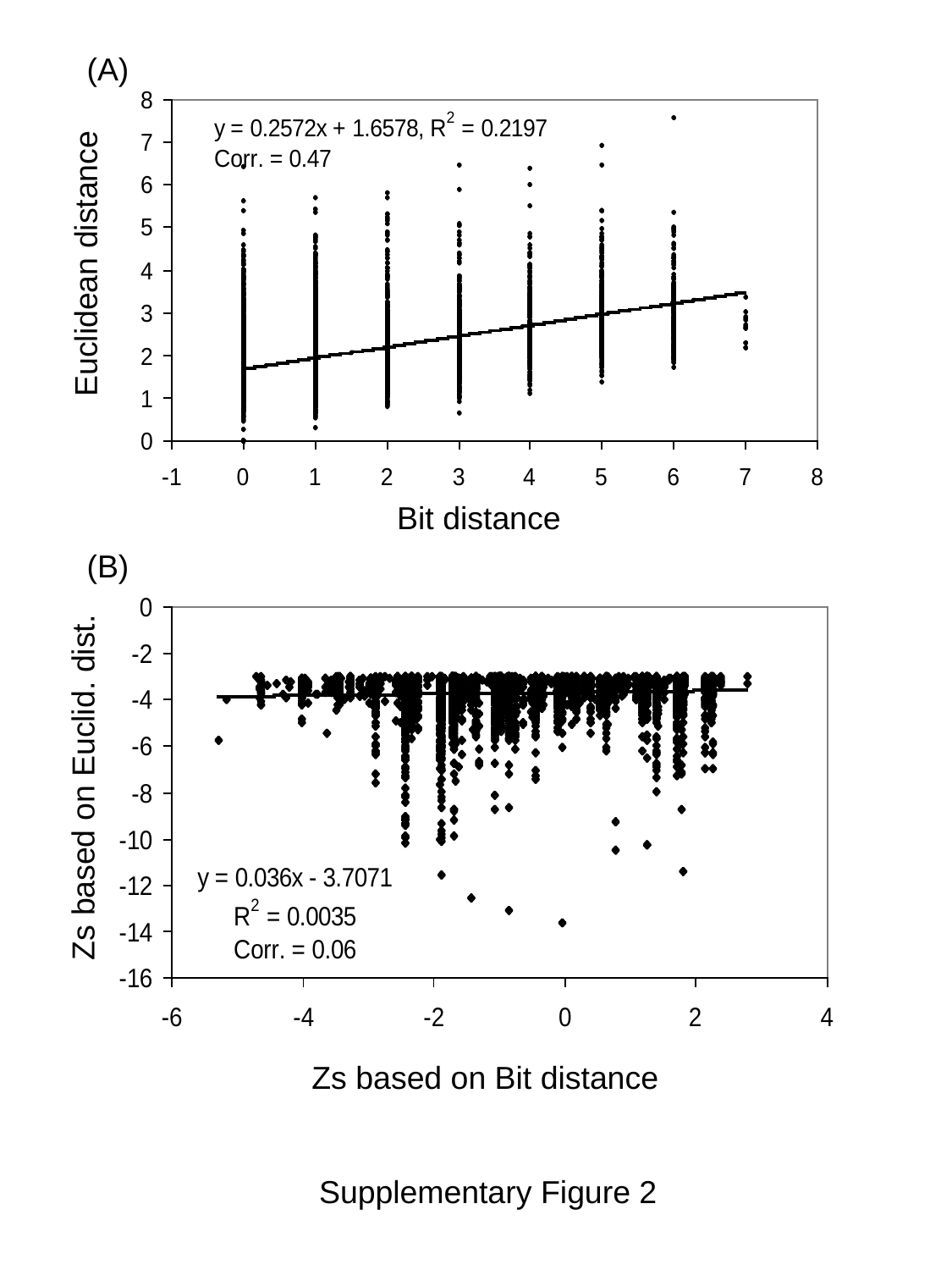

(A)
Euclidean distance
Bit distance
(B)
Zs based on Euclid. dist.
Zs based on Bit distance
Supplementary Figure 2
