# Supplementary material for: Predicting Protein Function with Hierarchical Phylogenetic Profiles: The Gene3D Phylo-Tuner Method Applied to Eukaryotic Genomes
Source: PLoS Comput Biol. 2007 Nov 30;3(11):e237. doi: 10.1371/journal.pcbi.0030237 (PMC2098864; doi:10.1371/journal.pcbi.0030237)
Supplement: Figure S4 — Profile comparison for the 4.10.900.10 cluster against the rest of the profiles in the extended matrix (see Example Predictions by Phylo-Tuner section). (A) Number of domain copies (y-axis) for the 1.10.880.10.2 cluster (thick blue line), for the cluster with the closest Ed (4.10.900.10, thick red line). The number of domain copies in different subclusters (S35) of the 4.10.900.10 Nt b-catenin binding subunit cluster are also shown. (B) Ed (y-axis) versus Bd (x-axis) for the comparison of 4.10.900.10 cluster against all other clusters. (230 KB PPT) [file pcbi.0030237.sg004.ppt]

## Slide 1
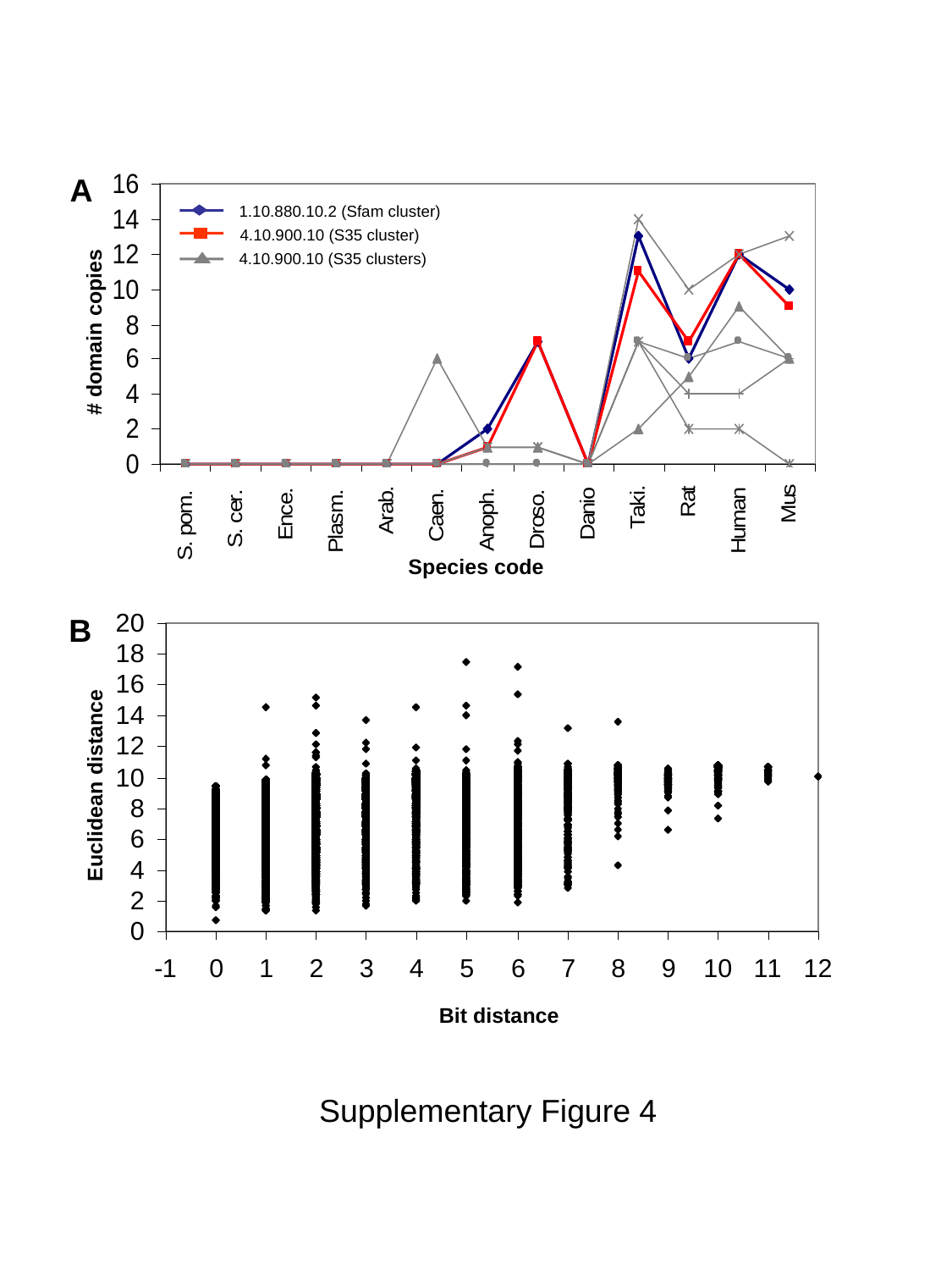

A
1.10.880.10.2 (Sfam cluster)
4.10.900.10 (S35 cluster)
4.10.900.10 (S35 clusters)
# domain copies
Species code
B
Euclidean distance
Bit distance
Supplementary Figure 4
