# Supplementary material for: Predicting Protein Function with Hierarchical Phylogenetic Profiles: The Gene3D Phylo-Tuner Method Applied to Eukaryotic Genomes
Source: PLoS Comput Biol. 2007 Nov 30;3(11):e237. doi: 10.1371/journal.pcbi.0030237 (PMC2098864; doi:10.1371/journal.pcbi.0030237)
Supplement: Figure S5 — For eukaryotic (A) and (B) and prokaryotic (C) and (D) profiles. (A,C) Eds for profile pairs (y-axis) versus the average sizes of the profiles (x-axis). (B,D) Logarithm of the Eds for the profile pairs (y-axis) versus logarithm of the average profile sizes (x-axis). (484 KB PPT) [file pcbi.0030237.sg005.ppt]

## Slide 1
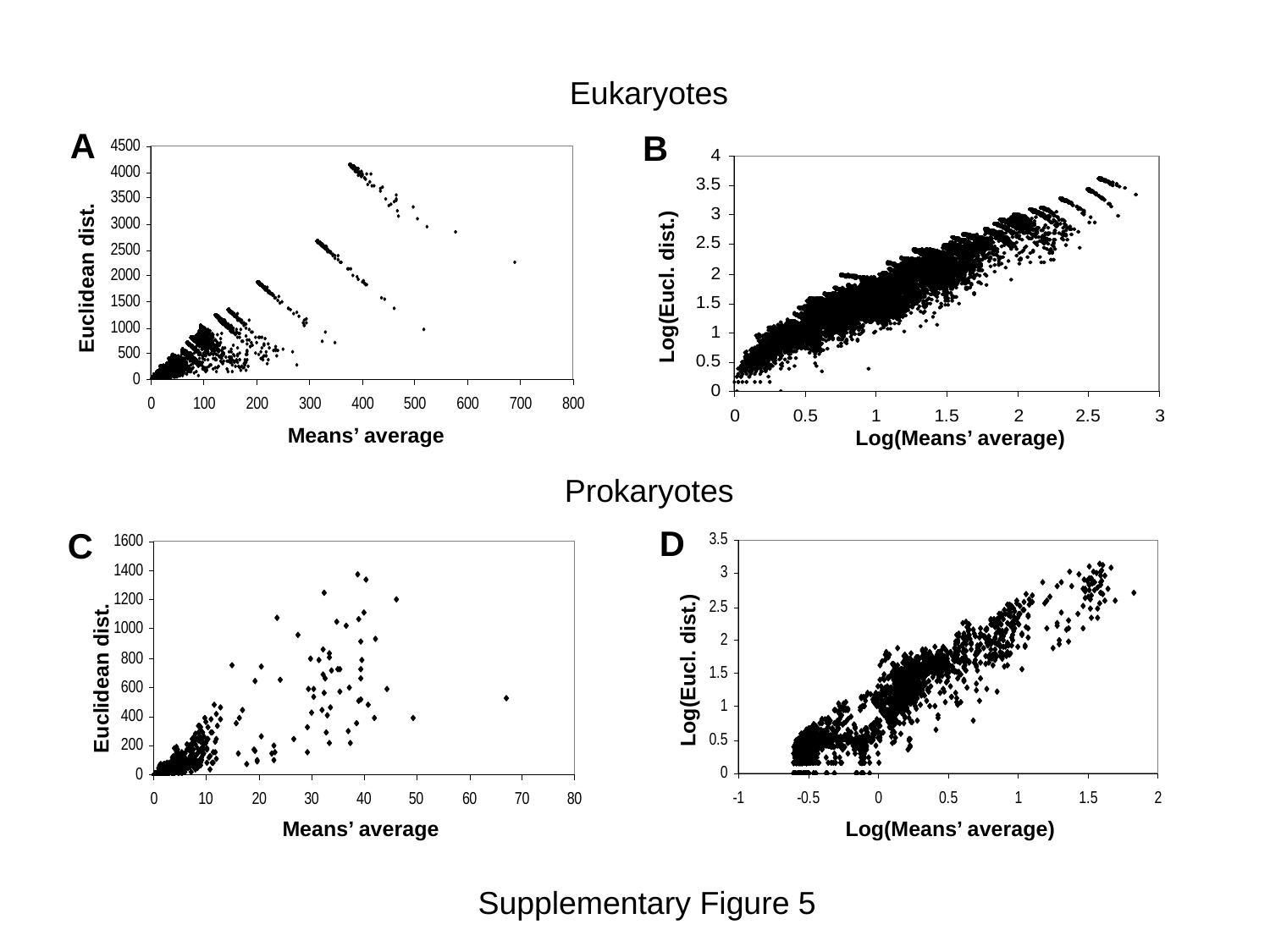

Eukaryotes
A
B
Euclidean dist.
Log(Eucl. dist.)
Means’ average
Log(Means’ average)
Prokaryotes
D
C
Log(Eucl. dist.)
Euclidean dist.
Means’ average
Log(Means’ average)
Supplementary Figure 5
