# Supplementary material for: Predicting Protein Function with Hierarchical Phylogenetic Profiles: The Gene3D Phylo-Tuner Method Applied to Eukaryotic Genomes
Source: PLoS Comput Biol. 2007 Nov 30;3(11):e237. doi: 10.1371/journal.pcbi.0030237 (PMC2098864; doi:10.1371/journal.pcbi.0030237)
Supplement: Figure S6 — The frequency distribution (left-hand y-axis) of TPs and FPs is plotted for the original analysis with homologous pairs (blue and light blue, respectively) and without (red and pink, respectively). The percent precision distribution (right-hand, y-axis) is shown for the analysis with homologous pairs (blue line) and without (red line) for all Zs value bins (x-axis for both distributions). Standard deviations are also indicated for the FP rates (vertical lines). Precision percentages were calculated based on the TP and FP frequencies for every Zs bin in the “no homologous pairs” sample, using for this new analysis the same profile matrix (with 3,721 protein clusters) and following the same procedure as described in the section Validation of Associated Profiles With GO Annotations and SS Scores (Material and Methods) and the section Assessment of Profile Similarity and Predicting Functionally Related Clusters (Results/Discussion). It can be seen in this plot that the TP rate in the “no homologous pairs” sample drops slightly from 0.041% to 0.035% in the highest Zs class (≤−3.5), compensated by a proportional decrease of the FP rate in the same Zs bin, giving virtually no variation in the precision ratio compared to the original analysis. For the remaining Zs bins, no significant differences are observed in the TP rate, the FP rate, or the precision values, demonstrating that no significant upward bias of the precision ratio is caused by the inclusion of homologous pairs in the analysis. (60 KB PPT) [file pcbi.0030237.sg006.ppt]

## Slide 1
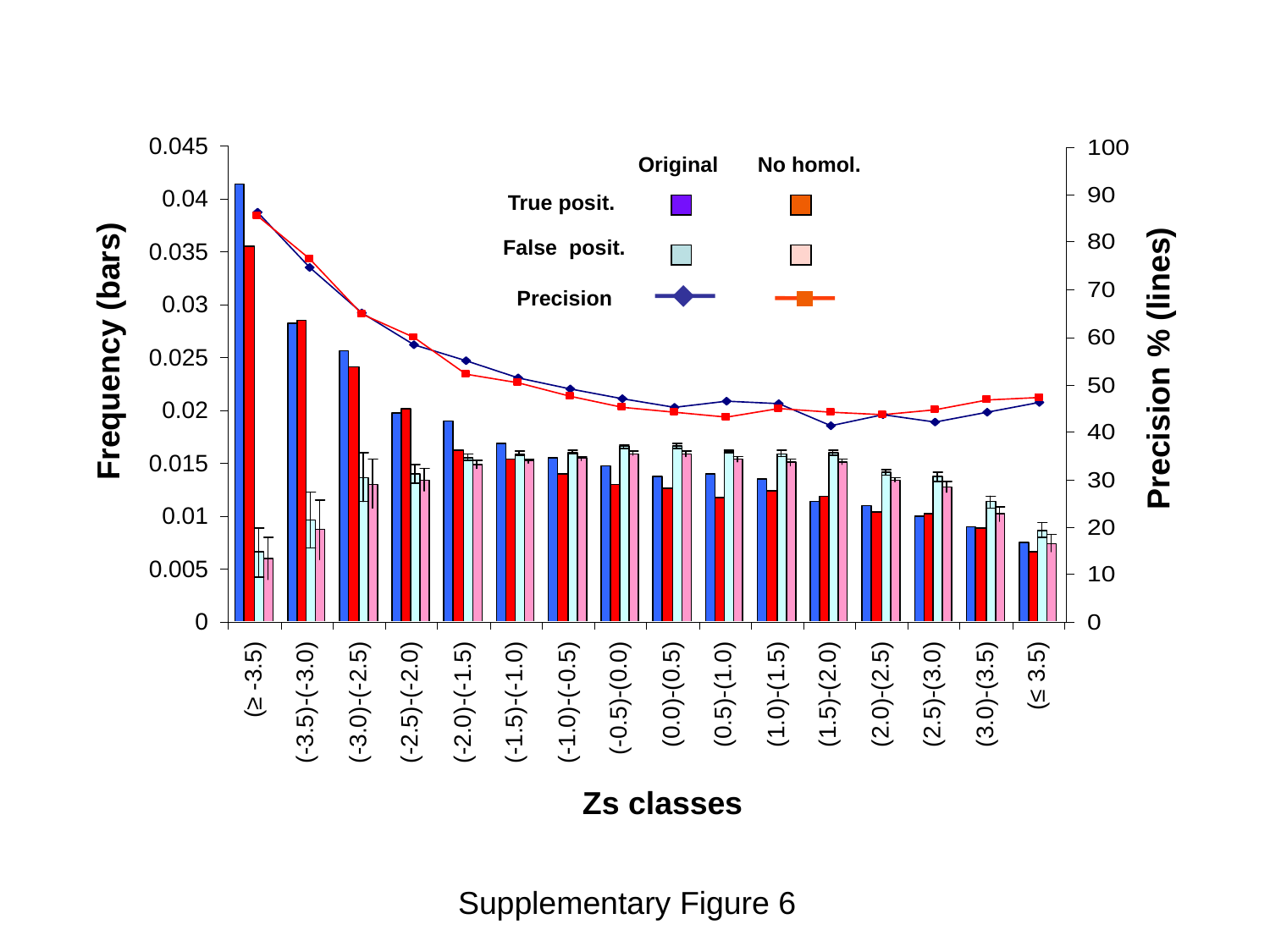

Original
No homol.
True posit.
Frequency (bars)
Precision % (lines)
False posit.
Precision
Zs classes
Supplementary Figure 6
