# Supplementary material for: Predicting Protein Function with Hierarchical Phylogenetic Profiles: The Gene3D Phylo-Tuner Method Applied to Eukaryotic Genomes
Source: PLoS Comput Biol. 2007 Nov 30;3(11):e237. doi: 10.1371/journal.pcbi.0030237 (PMC2098864; doi:10.1371/journal.pcbi.0030237)
Supplement: Figure S7 — Pairwise Ed (x-axis) distributions by percentage (y-axis). (A) The homologous (blue line) and the nonhomologous (red line) pairs. (B) Distributions for the clusters in the homologous 3.40.50.300 superfamily (blue line) against the rest of clusters in the matrix (red line). Using the same matrix of 3,721 protein clusters referenced above, the Ed distributions of homologous and nonhomologous pairs were calculated independently and compared between both sets (A). Homologous pairs count for only 6% of all pair comparisons, and these pairs therefore have a low statistical weight in the whole statistical analysis. From this comparison it can be seen that the homologous pairs show a very slight bias toward lower Eds. However, if the Ed distribution of homologous pairs from the superfamily 3.40.50.300 (a superfamily which is large enough for significant statistical comparison) is compared with the distance distribution for all the remaining 3.40.50.300 nonhomologous clusters (B), no significant difference is seen. Therefore, the likelihood of finding significant partners within or outside the superfamily are practically the same. These results indicate the possibility that homologous clusters could be co-evolving in a similar manner to nonhomologous pairs when a functional association between them is retained in evolution. For this reason, the co-evolution signal arising from the comparison of homologous profiles is retained within the Phylo-Tuner analysis. (61 KB PPT) [file pcbi.0030237.sg007.ppt]

## Slide 1
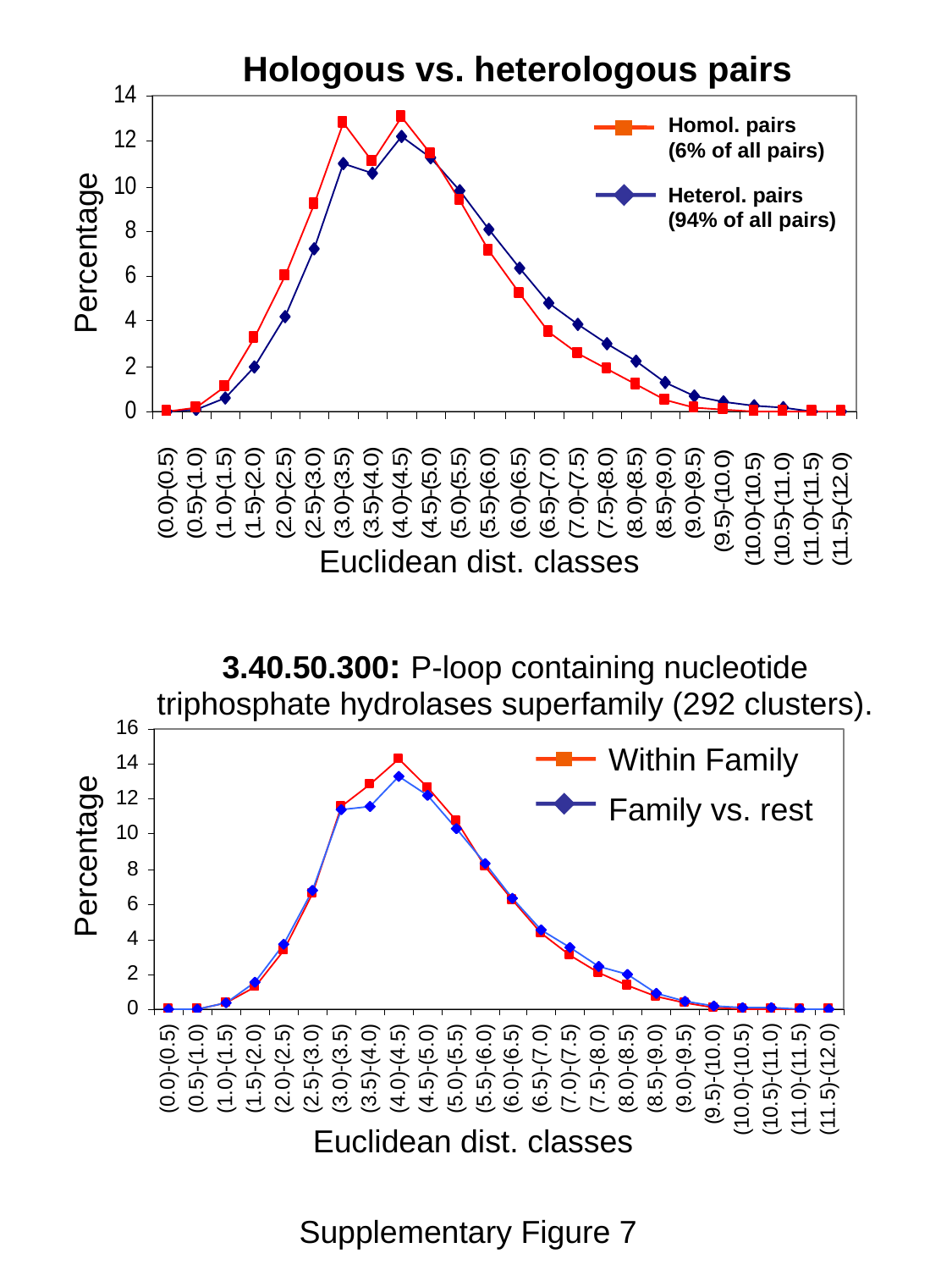

| Hologous vs. heterologous pairs |
| --- |
Homol. pairs
(6% of all pairs)
Percentage
Heterol. pairs
(94% of all pairs)
Euclidean dist. classes
| 3.40.50.300: P-loop containing nucleotide triphosphate hydrolases superfamily (292 clusters). |
| --- |
Within Family
Percentage
Family vs. rest
Euclidean dist. classes
Supplementary Figure 7
