# Supplementary material for: Predicting Protein Function with Hierarchical Phylogenetic Profiles: The Gene3D Phylo-Tuner Method Applied to Eukaryotic Genomes
Source: PLoS Comput Biol. 2007 Nov 30;3(11):e237. doi: 10.1371/journal.pcbi.0030237 (PMC2098864; doi:10.1371/journal.pcbi.0030237)
Supplement: Figure S8 — Percentage of profile pairs (y-axis) in each Ed bin (x-axis) for eukaryotic profiles with gene representation in six or more organisms (A) and in five or more organisms (B), for the real matrix (blue), the GS model (pink, GS), and for the PS model (yellow, PS). (C) Analysis of the increase in precision obtained by having at least six species in the profile. The plot on the right hand of (C) shows, for the smallest Ed bin, the percentage of profile pairs from the real matrix as TPs (TP5), and the percentage of profile pairs from the random models as FPs (FPps5 and FPgs5). In the left-hand plot of (C), the same is shown but for the six species matrix. (D) Precision values are estimated for the two different sources of FPs (PS and GS random models) and for the two different real matrices: five and six species. (64 KB PPT) [file pcbi.0030237.sg008.ppt]

## Slide 1
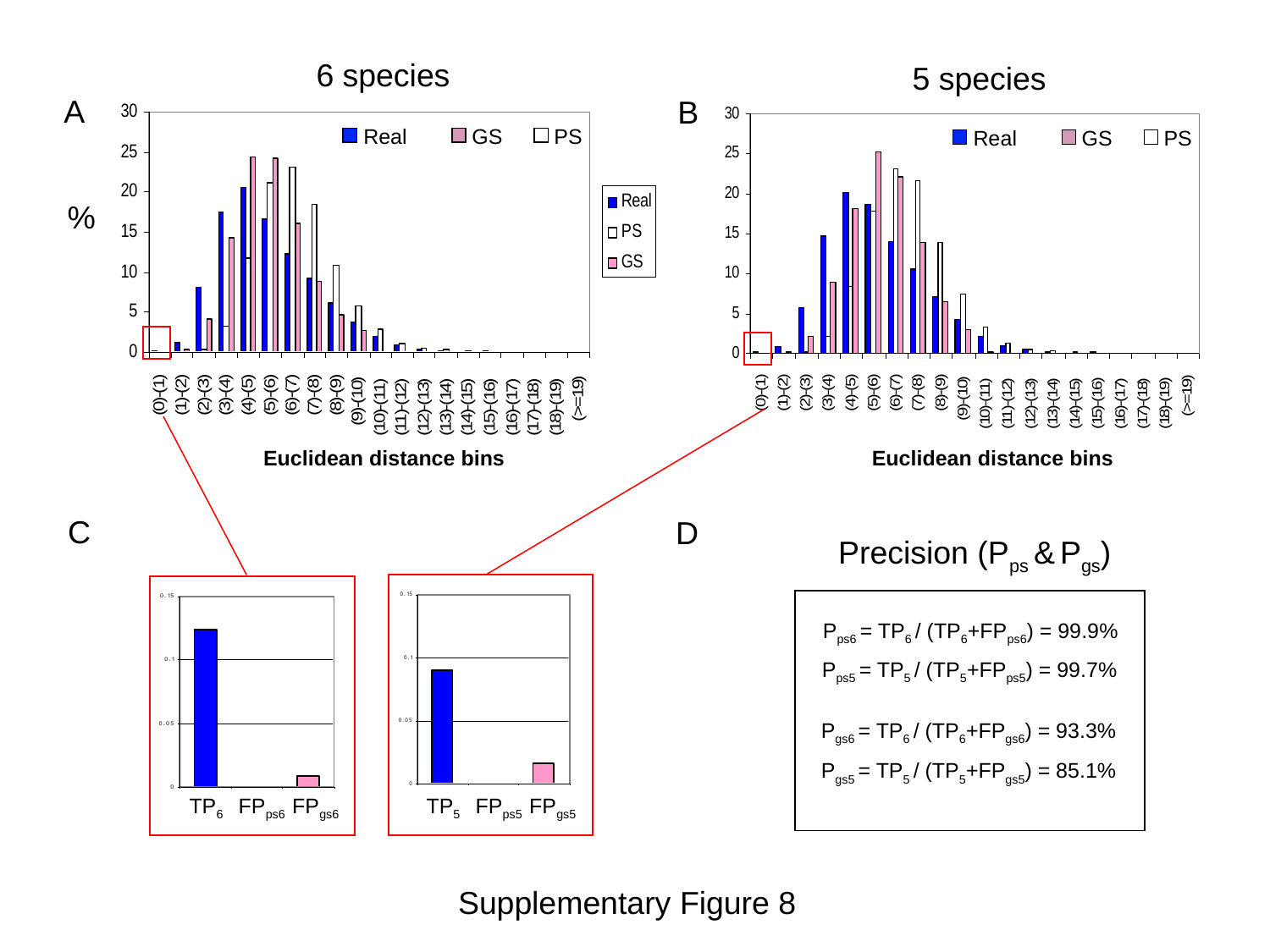

6 species
5 species
A
B
Real
GS
PS
Real
GS
PS
%
Euclidean distance bins
Euclidean distance bins
C
D
Precision (Pps & Pgs)
Pps6 = TP6 / (TP6+FPps6) = 99.9%
Pps5 = TP5 / (TP5+FPps5) = 99.7%
Pgs6 = TP6 / (TP6+FPgs6) = 93.3%
Pgs5 = TP5 / (TP5+FPgs5) = 85.1%
FPps6
FPgs6
TP6
FPps5
FPgs5
TP5
Supplementary Figure 8
