# Supplementary material for: Predicting Protein Function with Hierarchical Phylogenetic Profiles: The Gene3D Phylo-Tuner Method Applied to Eukaryotic Genomes
Source: PLoS Comput Biol. 2007 Nov 30;3(11):e237. doi: 10.1371/journal.pcbi.0030237 (PMC2098864; doi:10.1371/journal.pcbi.0030237)
Supplement: Table S1 — (36 KB DOC) [file pcbi.0030237.st001.doc]

| **Cluster 1** | **Cluster 2** | **Eucl/**  **Av.** | **Zs** | **N. sp. 1** | **N. sp. 2** | **Size 1** | **Size 2** | **Description 1** | **Description 2** |
| --- | --- | --- | --- | --- | --- | --- | --- | --- | --- |
| 2.60.40.840 | 4.10.400.10.25.2 | 0.6 | -3.67 | 8 | 7 | 61 | 61 | Arrestin (Nt domain) | Low-density Lipoprotein Receptor |
| 2.60.40.720 | 1.10.10.60.7.1 | 0.66 | -3.88 | 7 | 8 | 76 | 82 | Runt and p53 DNA-binding domain like | Homeodomain-like (DNA binding domain), hox gene-like cluster. |
| 1.10.510.10.13 | 1.10.10.10.18.1 | 0.68 | -4.29 | 7 | 7 | 94 | 94 | Eukaryotic protein Tyrosine kinases (Signal transduction) | ETS winged helix repressor DNA binding-like domain (Transcription Factor) |
| 1.10.30.10.6 | 4.10.400.10.25 | 0.7 | -4.54 | 8 | 7 | 107 | 111 | SOX (SRY-related HMG Transcritption Factor (DNA-binding protein) | Low-density Lipoprotein Receptor |
| 2.60.40.720 | 1.10.30.10.6.1.1 | 0.76 | -3.68 | 7 | 8 | 76 | 73 | Runt and p53 DNA-binding domain like | SOX (SRY-related HMG Transcritption Factor (DNA-binding protein) |
| 1.10.150.20 | 3.40.50.2060 | 0.81 | -3.69 | 12 | 13 | 79 | 79 | 5' to 3' exonuclease I-like, C-terminal domains | Syntaxin binding protein (Sec1 Family -Endocytosis/Exocytosis ) |
| 2.40.10.170 | 3.40.50.850 | 0.82 | -3.04 | 11 | 11 | 27 | 28 | ATP synthase alpha/beta family, beta-barrel domain (N-terminal domain) | Isochorismatase fiamily domain (Unknown Function) |
| 3.20.19.10 | 3.70.10.10 | 0.87 | -3.13 | 11 | 12 | 58 | 54 | Iron regulatory/Aconitase factor family, C-terminal RNA recognition domain | Proliferating cell nuclear antigen, N-terminal domain/ DNA polymerase processivity factor family (cell-cycle arrest) |
| 1.10.8.60 | 2.20.25.10 | 0.95 | -4.86 | 13 | 12 | 158 | 160 | ATPase family associated with various cellular activities (AAA) | Transcritption Factor (TFIIB zinc-binding-like domain) |
| 3.10.110.10.17 | 2.60.34.10.2.1.1.1 | 0.96 | -3.45 | 9 | 9 | 14 | 13 | Ubiquitin-conjugating enzyme domain. | Heat shock substrate binding domain of hsc-70 chaperone |

**Supplementary Table I. Ten examples of predicted cluster pairs with novel functional relationships.** From left to right: codes of cluster 1 and 2 in Gene3D; Euclidean distance divided by clusters’ average size; Zs value; N sp. 1 and 2 - number of species where clusters 1 and 2 are present; Size 1 and 2 - number of domain copies in Clusters 1 and 2; Description 1 and 2 – descriptive family name for clusters 1 and 2.
