# Supplementary material for: Predicting Protein Function with Hierarchical Phylogenetic Profiles: The Gene3D Phylo-Tuner Method Applied to Eukaryotic Genomes
Source: PLoS Comput Biol. 2007 Nov 30;3(11):e237. doi: 10.1371/journal.pcbi.0030237 (PMC2098864; doi:10.1371/journal.pcbi.0030237)
Supplement: Table S2 — (118 KB DOC) [file pcbi.0030237.st002.doc]

**Supplementary Table II.** Shows the 106 prokaryotic species used in the analysis.

| **Specie name** | **Filum** | **Taxa** |
| --- | --- | --- |
| Aeropyrum pernix | Archaea | Crenarchaeota |
| Pyrobaculum aerophilum | Archaea | Crenarchaeota |
| Sulfolobus solfataricus | Archaea | Crenarchaeota |
| Sulfolobus tokodaii | Archaea | Crenarchaeota |
| Archaeoglobus fulgidus | Archaea | Euryarchaeota |
| Halobacterium sp. NRC-1 | Archaea | Euryarchaeota |
| Methanocaldococcus jannaschii | Archaea | Euryarchaeota |
| Methanopyrus kandleri AV19 | Archaea | Euryarchaeota |
| Methanosarcina acetivorans C2A | Archaea | Euryarchaeota |
| Methanosarcina mazei Goe1 | Archaea | Euryarchaeota |
| Methanothermobacter thermautotrophicus str. | Archaea | Euryarchaeota |
| Pyrococcus abyssi | Archaea | Euryarchaeota |
| Pyrococcus furiosus DSM 3638 | Archaea | Euryarchaeota |
| Pyrococcus horikoshii | Archaea | Euryarchaeota |
| Thermoplasma acidophilum | Archaea | Euryarchaeota |
| Thermoplasma volcanium | Archaea | Euryarchaeota |
| Bifidobacterium longum NCC2705 | Bacteria | Actinobacteria |
| Corynebacterium efficiens YS-314 | Bacteria | Actinobacteria |
| Corynebacterium glutamicum ATCC 13032 | Bacteria | Actinobacteria |
| Mycobacterium leprae | Bacteria | Actinobacteria |
| Mycobacterium tuberculosis CDC1551 | Bacteria | Actinobacteria |
| Mycobacterium tuberculosis H37Rv | Bacteria | Actinobacteria |
| Streptomyces coelicolor A3(2) | Bacteria | Actinobacteria |
| Aquifex aeolicus | Bacteria | Aquificae |
| Chlamydia muridarum | Bacteria | Chlamydiae |
| Chlamydia trachomatis | Bacteria | Chlamydiae |
| Chlamydophila pneumoniae AR39 | Bacteria | Chlamydiae |
| Chlamydophila pneumoniae CWL029 | Bacteria | Chlamydiae |
| Chlamydophila pneumoniae J138 | Bacteria | Chlamydiae |
| Chlorobium tepidum TLS | Bacteria | Chlorobi |
| Nostoc sp. PCC 7120 | Bacteria | Cyanobacteria |
| Synechocystis sp. PCC 6803 | Bacteria | Cyanobacteria |
| Thermosynechococcus elongatus BP-1 | Bacteria | Cyanobacteria |
| Deinococcus radiodurans | Bacteria | Deinococcus-Thermus |
| Bacillus halodurans | Bacteria | Firmicutes |
| Bacillus subtilis | Bacteria | Firmicutes |
| Clostridium acetobutylicum | Bacteria | Firmicutes |
| Clostridium perfringens | Bacteria | Firmicutes |
| Clostridium tetani E88 | Bacteria | Firmicutes |
| Lactobacillus plantarum WCFS1 | Bacteria | Firmicutes |
| Lactococcus lactis subsp. lactis | Bacteria | Firmicutes |
| Listeria innocua | Bacteria | Firmicutes |
| Listeria monocytogenes EGD-e | Bacteria | Firmicutes |
| Mycoplasma genitalium | Bacteria | Firmicutes |
| Mycoplasma penetrans | Bacteria | Firmicutes |
| Mycoplasma pneumoniae | Bacteria | Firmicutes |
| Mycoplasma pulmonis | Bacteria | Firmicutes |
| Oceanobacillus iheyensis | Bacteria | Firmicutes |
| Staphylococcus aureus subsp. aureus Mu50 | Bacteria | Firmicutes |
| Staphylococcus aureus subsp. aureus MW2 | Bacteria | Firmicutes |
| Staphylococcus aureus subsp. aureus N315 | Bacteria | Firmicutes |
| Staphylococcus epidermidis ATCC 12228 | Bacteria | Firmicutes |
| Streptococcus agalactiae 2603V/R | Bacteria | Firmicutes |
| Streptococcus agalactiae NEM316 | Bacteria | Firmicutes |
| Streptococcus mutans UA159 | Bacteria | Firmicutes |
| Streptococcus pneumoniae R6 | Bacteria | Firmicutes |
| Streptococcus pneumoniae TIGR4 | Bacteria | Firmicutes |
| Streptococcus pyogenes M1 GAS | Bacteria | Firmicutes |
| Streptococcus pyogenes MGAS8232 | Bacteria | Firmicutes |
| Streptococcus pyogenes phage 315.6 | Bacteria | Firmicutes |
| Thermoanaerobacter tengcongensis | Bacteria | Firmicutes |
| Ureaplasma urealyticum | Bacteria | Firmicutes |
| Fusobacterium nucleatum subsp. nucleatum ATCC | Bacteria | Fusobacteria |
| Agrobacterium tumefaciens str. C58 (Cereon) | Bacteria | Proteobacteria |
| Agrobacterium tumefaciens str. C58 (U. | Bacteria | Proteobacteria |
| Bradyrhizobium japonicum | Bacteria | Proteobacteria |
| Brucella melitensis | Bacteria | Proteobacteria |
| Brucella suis 1330 | Bacteria | Proteobacteria |
| Buchnera aphidicola (Baizongia pistaciae) | Bacteria | Proteobacteria |
| Buchnera aphidicola str. Sg (Schizaphis | Bacteria | Proteobacteria |
| Buchnera sp. APS | Bacteria | Proteobacteria |
| Campylobacter jejuni | Bacteria | Proteobacteria |
| Caulobacter crescentus CB15 | Bacteria | Proteobacteria |
| Escherichia coli CFT073 | Bacteria | Proteobacteria |
| Escherichia coli K12 | Bacteria | Proteobacteria |
| Escherichia coli O157 H7 | Bacteria | Proteobacteria |
| Escherichia coli O157 H7 EDL933 | Bacteria | Proteobacteria |
| Haemophilus influenzae Rd | Bacteria | Proteobacteria |
| Helicobacter pylori 26695 | Bacteria | Proteobacteria |
| Helicobacter pylori J99 | Bacteria | Proteobacteria |
| Mesorhizobium loti | Bacteria | Proteobacteria |
| Neisseria meningitidis MC58 | Bacteria | Proteobacteria |
| Neisseria meningitidis Z2491 | Bacteria | Proteobacteria |
| Pasteurella multocida | Bacteria | Proteobacteria |
| Pseudomonas aeruginosa PAO1 | Bacteria | Proteobacteria |
| Pseudomonas putida KT2440 | Bacteria | Proteobacteria |
| Ralstonia solanacearum | Bacteria | Proteobacteria |
| Rickettsia conorii | Bacteria | Proteobacteria |
| Rickettsia prowazekii | Bacteria | Proteobacteria |
| Salmonella enterica subsp. enterica serovar | Bacteria | Proteobacteria |
| Salmonella typhimurium LT2 | Bacteria | Proteobacteria |
| Shewanella oneidensis MR-1 | Bacteria | Proteobacteria |
| Shigella flexneri 2a str. 301 | Bacteria | Proteobacteria |
| Sinorhizobium meliloti | Bacteria | Proteobacteria |
| Vibrio cholerae | Bacteria | Proteobacteria |
| Wigglesworthia brevipalpis | Bacteria | Proteobacteria |
| Xanthomonas axonopodis pv. citri str. 306 | Bacteria | Proteobacteria |
| Xanthomonas campestris pv. campestris str. ATCC | Bacteria | Proteobacteria |
| Xylella fastidiosa 9a5c | Bacteria | Proteobacteria |
| Xylella fastidiosa Temecula1 | Bacteria | Proteobacteria |
| Yersinia pestis | Bacteria | Proteobacteria |
| Yersinia pestis KIM | Bacteria | Proteobacteria |
| Borrelia burgdorferi | Bacteria | Spirochaetes |
| Leptospira interrogans serovar lai str. 56601 | Bacteria | Spirochaetes |
| Treponema pallidum | Bacteria | Spirochaetes |
| Thermotoga maritima | Bacteria | Thermotogae |
