# Supplementary material for: Predicting Protein Function with Hierarchical Phylogenetic Profiles: The Gene3D Phylo-Tuner Method Applied to Eukaryotic Genomes
Source: PLoS Comput Biol. 2007 Nov 30;3(11):e237. doi: 10.1371/journal.pcbi.0030237 (PMC2098864; doi:10.1371/journal.pcbi.0030237)
Supplement: Table S3 — (29 KB DOC) [file pcbi.0030237.st003.doc]

**Supplementary Table III.** Clustering of the 192,635 domain sequences annotated in the eukaryotic sample without applying any threshold. Number of clusters (second column) for each sequence identity level.

| **Identity Level** | **# of clusters** |
| --- | --- |
| **Sfam** | **1039** |
| **s30** | **41167** |
| **s35** | **48456** |
| **s40** | **56597** |
| **s50** | **70510** |
| **s60** | **85856** |
| **s70** | **102265** |
| **s80** | **120990** |
| **s90** | **142529** |
| **s95** | **157557** |
| **s100** | **192635** |
