# Supplementary material for: Predicting Protein Function with Hierarchical Phylogenetic Profiles: The Gene3D Phylo-Tuner Method Applied to Eukaryotic Genomes
Source: PLoS Comput Biol. 2007 Nov 30;3(11):e237. doi: 10.1371/journal.pcbi.0030237 (PMC2098864; doi:10.1371/journal.pcbi.0030237)
Supplement: Table S4 — (25 KB DOC) [file pcbi.0030237.st004.doc]

**Supplementary Table IV.** Number of functionally clusters pairs selected in each functional group dataset (figures in the matrix diagonals), and shared by the different datasets’ pairs (figures with %): Cell. comp. –Cellular components-; Biol. Proc. –Biological processes-; Mol. Func. –Molecular functions-.

|  | **Cell. comp.** | **Biol. proc.** | **Mol. func.** |
| --- | --- | --- | --- |
| **Cell. comp.** | 9,050 | 1,610 **(6%)** | 1,163 **(6%)** |
| **Biol. proc.** |  | 17,860 | 2,622 **(10%)** |
| **Mol. func.** |  |  | 9,346 |
