# Supplementary material for: Predicting Protein Function with Hierarchical Phylogenetic Profiles: The Gene3D Phylo-Tuner Method Applied to Eukaryotic Genomes
Source: PLoS Comput Biol. 2007 Nov 30;3(11):e237. doi: 10.1371/journal.pcbi.0030237 (PMC2098864; doi:10.1371/journal.pcbi.0030237)
Supplement: Text S1 — (52 KB DOC) [file pcbi.0030237.sd001.doc]

**Supplementary material:**

Supplementary Figure 1. Species (x-axis) ordered by their genome sizes (y-axis) in the eukaryotic a) and prokaryotic b) samples. Genome size is measured as the number of sequence domains found in the Gene3D database. Whilst the prokaryotic sample shows an almost continuous representation of genome size values, the eukaryotic sample shows a heterogeneous distribution with at least three different groups: Group 1, 2 and 3 in the a) plot.

Supplementary Figure 2. Comparison of Euclidean (Ed) and Bit (Bd) values. Comparison for the sample of significant predictions (Zs ≤ -3.0) provided by the Phylo-Tuner method. (A) Euclidean distance (y-axis) versus Bit distance (x-axis), and (B) Zs values based on Ed (y-axis) and Bd (x-axis) distributions. Trend line equations, R-squared values, and correlation coefficients are also indicated.

Supplementary Figure 3. Profile comparison of the 3.50.7.10 domain cluster against the rest of profiles in the extended matrix (see prediction examples section). a) Number of domain copies (y-axis) for the 3.90.640.10 cluster (thick blue line) is shown and for the cluster with the closest Euclidean distance (3.50.7.10 - CCT- thick red line). The number of domain copies in the different sub-clusters (S35) of the 3.50.7.10 CCT cluster are also shown. b) Euclidean distance (y-axis) versus the corresponding bit distance (x-axis) for the comparison of 3.50.7.10 cluster against all other clusters.

Supplementary Figure 4. Profile comparison for the 4.10.900.10 cluster against the rest of the profiles in the extended matrix (see prediction examples section). a) Number of domain copies (y-axis) for the 1.10.880.10.2 cluster (thick blue line), for the cluster with the closest Euclidean distance (4.10.900.10, thick red line). The number of domain copies in different sub-clusters (S35) of the 4.10.900.10 Nt b-catenin binding subunit cluster are also shown. b) Euclidean distance (y-axis) versus bit distance (x-axis) for the comparison of 4.10.900.10 cluster against all other clusters.

Supplementary Figure 5. Profile similarity score analysis. For eukaryotic -a) and b)- and prokaryotic -c) and d)- profiles: a) and c) Euclidean distances for profile pairs (y-axis) versus the average sizes of the profiles (x-axis); and b) and d) logarithm of the Euclidean distances for the profile pairs (y-axis) versus logarithm of the average profile sizes (x-axis).

Supplementary Figure 6. Comparison of the analysis with homologous pairs against the analysis without homologous pairs. The frequency distribution (left hand y-axis) of True Positives and False Positives is plotted for the original analysis with homologous pairs (blue and light-blue respectively) and without (red and pink respectively). The percent precision distribution (right hand, y-axis) is shown for the analysis with homologous pairs (blue line) and without (red line) for all Zs value bins (x-axis for both distributions). Standard deviations are also indicated for the False Positive rates (vertical lines). Precision percentages were calculated based on the True Positive and False Positive frequencies for every Zs bin in the “no homologous pairs” sample, using for this new analysis the same profile matrix (with 3,721 protein clusters) and following the same procedure as described in the section “Validation of Associated Profiles With GO Annotations and SS Scores” (Material and Methods) and the section “Assessment of Profile Similarity and Predicting Functionally Related Clusters” (Results and Discussion). It can be seen in this plot that the True Positive (TP) rate in the “no homologous pairs” sample drops slightly from 0.041% to 0.035% in the highest Zs class (≤ -3.5), compensated by a proportional decrease of the False Positive (FP) rate in the same Zs bin, giving virtually no variation in the precision ratio compared to the original analysis. For the remaining Zs bins no significant differences are observed in the TP rate, the FP rate or the precision values, demonstrating that no significant upward bias of the precision ratio is caused by the inclusion of homologous pairs in the analysis.

Supplementary Figure 7. Pair-wise Euclidean distance (x-axis) distributions by percentage (y-axis). (A) the homologous (blue line) and the non-homologous (red line) pairs; (B) distributions for the clusters in the homologous 3.40.50.300 superfamily (blue line) against the rest of clusters in the matrix (red line). Using the same matrix of 3,721 protein clusters referenced above, the Euclidean distance distributions of homologous and non-homologous pairs were calculated independently and compared between both sets (plot A). Homologous pairs count for only 6% of all pair comparisons and these pairs therefore have a low statistical weight in the whole statistical analysis. From this comparison it can be seen that the homologous pairs show a very slight bias towards lower Euclidean distances. However, if the Euclidean distance distribution of homologous pairs from the superfamily 3.40.50.300 (a superfamily which is large enough for significant statistical comparison) is compared with the distance distribution for all the remaining 3.40.50.300 non-homologous clusters (plot B), no significant difference is seen. Therefore, the likelihood of finding significant partners within or outside the superfamily are practically the same. These results indicate the possibility that homologous clusters could be co-evolving in a similar manner to non-homologous pairs when a functional association between them is retained in evolution. For this reason the co-evolution signal arising from the comparison of homologous profiles is retained within the Phylo-Tuner analysis.

Supplementary Figure 8. Percentage of profile pairs (y-axis) in each Euclidean distance bin (x-axis) for eukaryotic profiles with gene representation in 6 or more organisms a) and in 5 or more organisms b), for the real matrix (blue), the genome shuffling model (pink, GS), and for the profile shuffling model (yellow, PS). c) analysis of the increase in precision obtained by having at least 6 species in the profile. The plot on the right hand of c) shows, for the smallest Euclidean distance bin, the percentage of profile pairs from the real matrix as true positives (TP5), and the percentage of profile pairs from the random models as false positives (FPps5 and FPgs5). In the left hand plot of c), the same is shown but for the 6 species matrix. And in d), precision values are estimated for the two different sources of false positives (PS and GS random models) and for the two different real matrices: 5 and 6 species.

**Supplementary Text 1:** Detailed bibliographic analysis of the ten pairs selected for Supplementary Table I**.** For three of the pairs it has not been possible to find any evidence from the literature supporting the putative functional associations, while for the remaining seven pairs the bibliographic study has provided information that supports the clusters’ functional links. The literature analysis for the ten examples is reported below:

Pair: 2.60.40.840 - 4.10.400.10.25.2

2.60.40.840 arrestin domain. Arrestins comprise a family of closely-related proteins, including beta-arrestin-1 and -2, which regulate the function of beta-adrenergic receptors by binding to their phosphorylated forms, impairing their capacity to activate G(S) proteins. Arrestins have also been implicated in the endocytosis of receptors and cross talk with other signalling pathways (Gurevich et al. 2006; Buchanan et al. 2006).

4.10.400.10.25.2: The low density lipoprotein (LDL) receptor protein binds LDL and transports it into cells by endocytosis. Low density lipoprotein (LDL) is the major cholesterol-carrying lipoprotein of plasma (May et al. 2007). There is some experimental evidence that functionally relates the Arrestins with the LDL receptor proteins (Wu et al. 2003).

Pair: 2.60.40.720 – 1.10.10.60.7.1:

2.60.40.720 corresponds to the Runt and p53-like domains. [Drosophila melanogaster](http://www.ncbi.nlm.nih.gov/Taxonomy/Browser/wwwtax.cgi?id=7227) segmentation gene runt is very similar to the acute myeloid leukemia (AML1) gene and the mouse transcription factor PEBP2 alpha subunit gene. The protein (known as acute myeloid leukemia 1 protein, oncogene AML-1) binds to the core site, 5'-pygpyggt-3', of a number of enhancers and promoters (Miyoshi et al., 1995). P53 domain is found in p53 transcription factors, where it is responsible for DNA-binding. These transcription factors play diverse roles in the regulation of cellular functions: the p53 tumour suppressor upregulates the expression of genes involved in cell cycle arrest and apoptosis (Sutcliffe et al., 2003)

1.10.10.60.7.1 corresponds to a homeobox domain, hox gene cluster. This domain was first identified in a number of drosophila homeotic and segmentation proteins, but it is now known to be well-conserved in many other animals, including vertebrates (Gehring, 1992). Hox genes encode homeodomain-containing transcriptional regulators that operate differential genetic programs along the anterior-posterior axis of animal bodies [PUBMED:12445403](http://www.ncbi.nlm.nih.gov:80/entrez/query.fcgi?cmd=Retrieve&db=PubMed&dopt=Abstract&list_uids=12445403). The domain binds DNA through a helix-turn-helix (HTH) structure. The 1.10.10.60.7.1 cluster includes NK-like and T-cell leukemia homeobox related genes.

There are various clues in the literature that relate the homebox genes in hematopoiesis and leukemogenesis, including acute myeloid leukemia (Shimamoto et al., 1998), and p53 with NK-like homeobox genes (Di Stefano et al., 2004; Lei et al., 2006).

Pair: 1.10.510.10.13 – 1.10.10.10.18.1

1.10.510.10.13 is a cluster of a tyrosine kinase domain proteins that mediate the response of eukaryotic cells to external stimuli by phosphorylation of hydroxyamino acids. The 1.10.510.10.13 cluster is represented by the ABL2, FRK, or CSF-like genes.

1.10.10.10.18.1 cluster corresponds to the ETS transcription factor domain. ETS family members have a highly conserved 85-amino acid ETS domain that binds purine-rich DNA sequences (PFAM data base code: PF00178; Ets). The 1.10.10.10.18.1 ETS domain cluster is represented amongst others by the FEV, ERF(ETS2), ELK, ELF, ETV like protein families.

There are numerous studies that relate members from these two domain clusters: ETV6-ABL Griesinger et al, 2002), FRK-ETV6 (Hosoya et al., 2005); ETS1–FLK1 (Elvert et al., 2003); ELF4 - CSF1: Yao et al., 2007); ETS2-CSF (Lu et al., 2003); ELK1–CSF1 (Yao GQ et al., 2005).

Pair: 1.10.30.10.6 – 4.10.400.10.25

1.10.30.10.6 cluster is a DNA-binding HMG (high mobility group) domain of the SOX (SRY-related HMG-box) family of transcription factors involved in the regulation of embryonic development and in the determination of the cell fate (Lefebvre et al., 2007).

As described above, the 4.10.400.10.25 cluster corresponds to the low density lipoprotein (LDL) receptor protein. Although there are only a few clues about the functional relationship between these two domains, it seems likely that they are both associated with chondrogenesis (Kirton et al., 2007).

Pair: 2.60.40.720 - 1.10.30.10.6.1.1

As described above, 2.60.40.720 corresponds to the Runt and p53-like domains, whilst the 1.10.30.10.6.1.1 cluster is a DNA-binding HMG (high mobility group) domain cluster of the SOX (SRY-related HMG-box) family of transcription factors.

There appears to be even stronger evidence of a functional relationship between the Sox and Runt domain families in skeletogenesis and osteogenesis (Noda et al., 1998; Zhou et al., 2006; Zou et al., 2006). There are also some clues about a possible relationship between Sox genes and p53 in the regulation of the IEX-1 gene that controls cell growth and apoptosis. (Im et al., 2002).

Pair: 1.10.150.20 – 3.40.50.2060:

1.10.150.20 cluster is an exonuclease 1-like domain present amongst other in relatives to the FEN1 (flap structure-specific endonuclease 1), DNTT (deoxynucleotidyltransferase), and polymerase (DNA directed – lambda) protein families. 3.40.50.2060 cluster corresponds to the Sec1 domain family involves in vesicle transport. Currently, there is no obvious evidence of a functional connection between these two domain clusters, in the literature.

Pair: 2.40.10.170 – 3.40.50.850

2.40.10.170 is the N_terminal domain of the ATP synthase alpha/beta family which are membrane-bound enzyme complexes/ion transporters that combine ATP synthesis and/or hydrolysis with the transport of protons across a membrane (PFAM database code: PF02874). 3.40.50.850 is a Isochorismatase family domain. Isochorismatase, also known as 2,3 dihydro-2,3 dihydroxybenzoate synthase catalyses the conversion of isochorismate, in the presence of water, to 2,3-dihydroxybenzoate and pyruvate. However, little is known about the functional diversification and roles of these enzymes in eukaryotes and there is no evidence of a functional relationship between these two protein domains in literature.

Pair: 3.20.19.10 – 3.70.10.10

3.20.19.10 is the C-terminal domain cluster of the aconitase enzyme. The 3.20.19.10 domain cluster is specifically related to the C-terminal domain of the iron regulatory element binding protein 1 (IREB1). IREB1 is a cytosolic protein which binds to iron-responsive elements (IREs). IREs are stem-loop structures found in the 5' UTR of ferritin mRNA, and in the 3' UTR of transferrin receptor mRNA. The iron-induced binding to the IRE results in repression of translation of ferritin mRNA, and inhibition of degradation of the otherwise rapidly degrading transferrin receptor mRNA. Thus, IREB1 plays a central role in cellular iron homeostasis. Iron acquisition is a fundamental requirement for many aspects of life, but excess iron may result in formation of free radicals that damage DNA (Cairo et al., 2002).

3.70.10.10 is a domain cluster required for transient cell-cycle arrests and transcriptional induction of DNA repair in response to DNA damage. This domain is specifically present in the PCNA -proliferating cell nuclear antigen- and RAD genes as RAD9 and RAD1 -also known as Rec1- proteins involved in cell cycle checkpoint of DNA damage or incomplete DNA replication (Niida & Nakanishi, 2005, Helt et al., 2005).

There is a clear functional relationship between iron regulation and cell cycle checkpoint proteins in the following studies - Philpott et al, 1998; Gazitt et al., 2001.

Pair: 1.10.8.60 – 2.20.25.10

There are no clear functional relationships in the literature between the 1.10.8.60 ATPase domain, which is associated with various cellular activities, and the 2.20.25.10 Transcritption Factor TFIIB-like zinc-binding domain.

Pair: 3.10.110.10.17 – 2.60.34.10.2.1.1.1

3.10.110.10.17 is the ubiquitin-conjugating E2 enzyme domain related to the UBE2W-like proteins, while 2.60.34.10.2.1.1.1 is the heat-shock 70 domain of the HSPA9P-like proteins. Despite the fact that there is no direct evidence in the literature supporting a functional relationship between the UBE2W and HSPA9P proteins, the functional involvement of Hsc70 chaperone in ubiquitin-dependent degradation is well supported and documented (Bercovich et al., 1997; Esser et al., 2004).

**References:**

Alonso CR (2002) Hox proteins: sculpting body parts by activating localized cell death. Curr Biol. 12: R776-R778.

Bercovich B, Stancovski I, Mayer A, Blumenfeld N, Laszlo A, Schwartz AL, Ciechanover A (1997) Ubiquitin-dependent degradation of certain protein substrates in vitro requires the molecular chaperone Hsc70. J Biol Chem. 272: 9002-9010.

Buchanan FG, DuBois RN (2006) Emerging roles of beta-arrestins. Cell Cycle. 5: 2060-2063.

Cairo G, Recalcati S, Pietrangelo A, Minotti G (2002) The iron regulatory proteins: targets and modulators of free radical reactions and oxidative damage. Free Radic Biol Med. 32: 1237-1243.

Di Stefano V, Rinaldo C, Sacchi A, Soddu S, D'Orazi G (2004) Homeodomain-interacting protein kinase-2 activity and p53 phosphorylation are critical events for cisplatin-mediated apoptosis. Exp Cell Res. 293: 311-320.

Elvert G, Kappel A, Heidenreich R, Englmeier U, Lanz S, Acker T, Rauter M, Plate K, Sieweke M, Breier G, Flamme I (2003 ) Cooperative interaction of hypoxia-inducible factor-2alpha (HIF-2alpha) and Ets-1 in the transcriptional activation of vascular endothelial growth factor receptor-2 (Flk-1). J Biol Chem. 278: 7520-7530.

Esser C, Alberti S, Hohfeld J (2004) Cooperation of molecular chaperones with the ubiquitin/proteasome system. Biochim Biophys Acta. 1695: 171-188.

Gazitt Y, Reddy SV, Alcantara O, Yang J, Boldt DH (2001) A new molecular role for iron in regulation of cell cycling and differentiation of HL-60 human leukemia cells: iron is required for transcription of p21(WAF1/CIP1) in cells induced by phorbol myristate acetate. J Cell Physiol. 187: 124-135.

Gehring WJ (1992) The homeobox in perspective. Trends Biochem Sci. 17: 277-280

Griesinger F, Janke A, Podleschny M, Bohlander SK (2002) Identification of an ETV6-ABL2 fusion transcript in combination with an ETV6 point mutation in a T-cell acute lymphoblastic leukaemia cell line. Br J Haematol. 119: 454-458.

Gurevich EV, Gurevich VV (2006) Arrestins: ubiquitous regulators of cellular signaling pathways. Genome Biol. 7: 236.

Helt CE, Wang W, Keng PC, Bambara RA (2005) Evidence that DNA damage detection machinery participates in DNA repair. Cell Cycle. 4: 529-532.

Hosoya N, Qiao Y, Hangaishi A, Wang L, Nannya Y, Sanada M, Kurokawa M, Chiba S, Hirai H, Ogawa S (2005) Identification of a SRC-like tyrosine kinase gene, FRK, fused with ETV6 in a patient with acute myelogenous leukemia carrying a t(6;12)(q21;p13) translocation. Genes Chromosomes Cancer 42: 269-279.

Im HJ, Pittelkow MR, Kumar R (2002) Divergent regulation of the growth-promoting gene IEX-1 by the p53 tumor suppressor and Sp1. J Biol Chem. 277: 14612-14621.

Jiang A, Yu C, Zhang P, Chen W, Liu W, Hu X, Zhang J (2006) Free Full Text p53 overexpression represses androgen-mediated induction of NKX3.1 in a prostate cancer cell line. Exp Mol Med. 38: 625-633.

Kirton JP, Crofts NJ, George SJ, Brennan K, Canfield AE (2007) Wnt/beta-catenin signalling stimulates chondrogenic and inhibits adipogenic differentiation of pericytes: potential relevance to vascular disease? Circ Res. 101: 581-589.

Lefebvre V, Dumitriu B, Penzo-Mendez A, Han Y, Pallavi B (2007) Control of cell fate and differentiation by Sry-related high-mobility-group box (Sox) transcription factors. Int J Biochem Cell Biol. Jun 6; [Epub ahead of print].

Lei Q, Jiao J, Xin L, Chang CJ, Wang S, Gao J, Gleave ME, Witte ON, Liu X, Wu H (2006) NKX3.1 stabilizes p53, inhibits AKT activation, and blocks prostate cancer initiation caused by PTEN loss. Cancer Cell. 9: 367-378.

Lu Z, Kim KA, Suico MA, Uto A, Seki Y, Shuto T, Isohama Y, Miyata T, Kai H (2003) ETS2 is involved in protein kinase C-activated expression of granulocyte-macrophage colony-stimulating factor in human non-small lung carcinoma cell line, A549. Biochem Biophys Res Commun. 303: 190-195.

May P, Woldt E, Matz RL, Boucher P (2007) The LDL receptor-related protein (LRP) family: an old family of proteins with new physiological functions. Ann Med. 39: 219-228.

Miyoshi H, Ohira M, Shimizu K, Mitani K, Hirai H, Imai T, Yokoyama K, Soeda E, Ohki M (1995) Alternative splicing and genomic structure of the AML1 gene involved in acute myeloid leukemia. Nucleic Acids Res. 23: 2762-2769.

Niida H, Nakanishi M (2006) DNA damage checkpoints in mammals. Mutagenesis. 21: 3-9.

Noda M, Nifuji A, Tuji K, Furuya K, Ichiro S, Asou Y, Kawaguchi N, Yamachita K (1998) Transcription factors and osteoblasts. Front Biosci. 3: d817-d820.

Philpott CC, Rashford J, Yamaguchi-Iwai Y, Rouault TA, Dancis A, Klausner RD (1998) Cell-cycle arrest and inhibition of G1 cyclin translation by iron in AFT1-1(up) yeast. EMBO J. 17: 5026-5036.

Shimamoto T, Ohyashiki K, Toyama K, Takeshita K (1998 ) Homeobox genes in hematopoiesis and leukemogenesis. Int J Hematol. 67: 339-350.

Sutcliffe JE, Korenjak M, Brehm A (2003 ) Tumour suppressors--a fly's perspective. Eur J Cancer. 39: 1355-1362.

Wu JH, Peppel K, Nelson CD, Lin FT, Kohout TA, Miller WE, Exum ST, Freedman NJ (2003) The adaptor protein beta-arrestin2 enhances endocytosis of the low density lipoprotein receptor. J Biol Chem. 278: 44238-44245.

Yao GQ, Itokawa T, Paliwal I, Insogna K (2005) CSF-1 induces fos gene transcription and activates the transcription factor Elk-1 in mature osteoclasts. Calcif Tissue Int. 76: 371-378.

Yao JJ, Liu Y, Lacorazza HD, Soslow RA, Scandura JM, Nimer SD, Hedvat CV (2007) Tumor promoting properties of the ETS protein MEF in ovarian cancer. Oncogene. 26: 4032-4037.

Zhou G, Zheng Q, Engin F, Munivez E, Chen Y, Sebald E, Krakow D, Lee B (2006 ) Dominance of SOX9 function over RUNX2 during skeletogenesis. Proc Natl Acad Sci U S A. 103: 19004-19009.

Zou L, Zou X, Li H, Mygind T, Zeng Y, Lu N, Bunger C (2006) Molecular mechanism of osteochondroprogenitor fate determination during bone formation. Adv Exp Med Biol. 585: 431-441.
